# Ruthenocenyl and 1‑Adamantyl Paclitaxel Analogs Disrupt the Balance between βIII- and βIVa-Tubulin and Inhibit the Growth and Invasiveness of Colon Cancer

**DOI:** 10.1021/acs.jmedchem.5c01147

**Published:** 2025-09-04

**Authors:** Wojciech M. Ciszewski, Karolina Kowalczyk, Andrzej Błauż, Wojciech Ciesielski, Piotr Hogendorf, Beata Smolarz, Waldemar Wagner, Anna Wieczorek-Błauż, Błażej Michał Rychlik, Hanna Romanowicz, Adam Durczyński, Katarzyna Sobierajska, Damian Plażuk

**Affiliations:** † Department of Molecular Cell Mechanisms, 37808 Medical University of Lodz, Mazowiecka 6/8, Lodz 92-215, Poland; ‡ Laboratory of Molecular Spectroscopy, Department of Organic Chemistry, Faculty of Chemistry, University of Lodz, Tamka 12, Lodz 91-403, Poland; § Centre for Digital Biology and Biomedical Science - Biobank Lodz, Faculty of Biology and Environmental Protection, University of Lodz, ul. Pomorska 141/143, Łódź 90-236, Poland; ∥ Department of General and Transplant Surgery, Medical University of Lodz, Kopcinskiego 22, Lodz 90-419, Poland; ⊥ Laboratory of Cancer Genetics, Department of Pathology, 37800 Polish Mother’s Memorial Hospital Research Institute, Rzgowska 281/289, Lodz 93-338, Poland; # Laboratory of Cellular Immunology, 111423 Institute of Medical Biology PAS, Lodowa 106, Lodz 93-232, Poland; ∇ Department of Organic Chemistry, Faculty of Chemistry, University of Lodz, Tamka 12, Lodz 91-403, Poland

## Abstract

Taxanes are widely used anticancer agents that stabilize microtubules and arrest cell division. However, their efficacy in colon cancer is limited by the chemoresistance associated with βIII-tubulin (TUBB3) upregulation. Herein, ferrocenyl, ruthenocenyl, and 1-adamantyl analogs of paclitaxel were synthesized and biologically evaluated. All compounds exhibited significantly higher cytotoxicity than paclitaxel, with IC_50_ values in the nanomolar range. Ruthenocenyl and 1-adamantyl analogs effectively inhibited both growth and invasiveness of colon cancer cells. These effects correlated with altered tubulin isoform expression (downregulation of βIII-tubulin and upregulation of βIVa-tubulin) were associated with modulation of the focal adhesion complex. Specifically, changes in microtubule interactions with the integrin-linked kinase–integrin−β1 axis contributed to a reduced invasive potential. The unique properties of these analogs suggest their potential for dual-action therapy combining tumor growth inhibition with metastasis prevention.

## Graphical abstract

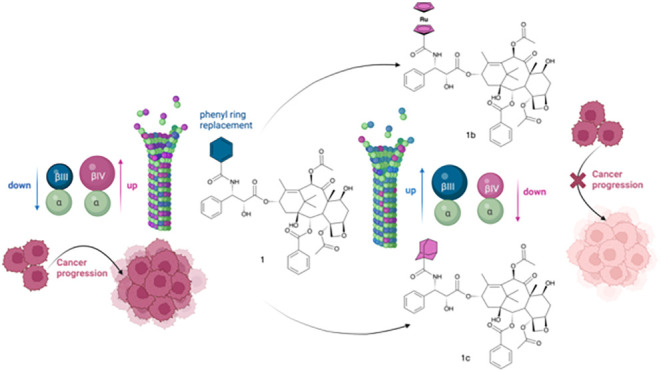

## Introduction

Microtubules, dynamic heteropolymers formed by α-tubulin (TUBA) and β-tubulin (TUBB) dimers,^1^ play crucial roles in eukaryotic cells by participating in cytoskeleton formation and are responsible for maintaining cell shape and structure. Furthermore, microtubules are indispensable for cell movement, intracellular trafficking of organelles, and primarily, cell division.^2–4^ Therefore, microtubules are popular targets in cancer therapy.^5^ Numerous natural and synthetic or semisynthetic compounds are known to be microtubule poisons that bind to one of the binding sites identified thus far, namely, laulimalide, taxane/epothilone, vinca alkaloid, colchicine, maytansine, and pironetine.^6^ These compounds either stabilize or destabilize microtubules, thus affecting their polymerization dynamics and leading to cell proliferation blockade.^7^ Paclitaxel **1**, a diterpenoid isolated from the Pacific yew (*Taxus brevifolia*), and its semisynthetic analogs docetaxel and cabazitaxel (collectively referred to as taxanes) are extensively employed for the treatment of breast, ovarian, prostate, and gastric cancers; non-small-cell lung cancer; and Kaposi’s sarcoma.^8,9^ Taxanes act as microtubule stabilizers and bind to specific sites of β-tubulin, which promotes the formation of distinct microtubule bundles and inhibits spindle formation, therefore leading to mitotic arrest and ultimately cell death.^10,11^ Microtubule polymerization/depolymerization is strictly regulated by the composition of the tubulin subunit (nine α-tubulin and eight β-tubulin isotypes).^12^ Despite the high anticancer potency of taxanes, their use is limited because of two main reasons: (i) a lack of selectivity toward cancer over noncancerous cells, which results in the high systemic toxicity of taxanes, and (ii) reduced or even completely abolished paclitaxel activity caused by taxane resistance.^13–15^

Resistance to taxanes is complex and involves multifunctional mechanisms comprising numerous pathways and genes that function alone or as a set of factors.^16–20^ This resistance can be nonspecific (i.e., multidrug resistance [MDR], which could be attributed to enhanced phase I and phase II enzyme activity, elevated expression of nonspecific drug efflux pumps such as ABCB1, or altered expression of drug influx transporters) or specific for the mode of action (associated with tubulin). One of the key action-specific mechanisms is the overexpression of βIII-tubulin, which is linked to the enhanced dynamicity of microtubules and their decreased ability to interact with taxanes owing to differences in their amino acid sequence.^19,21,22^ Furthermore, a reduction in microtubule dynamics increases the sensitivity of cancer cells to taxane treatment.^23,24^ Hence, as the polymerization/depolymerization dynamics of microtubules are altered by their composition, it is an essential parameter in taxane resistance.^25–27^ Studies have reported alterations in tubulin isotypes in cancer, and these changes are the main reason for the poor response to chemotherapy and poor prognosis.^22^ Alterations in β-tubulin in cancers are correlated with the decreased effectiveness of anticancer therapy.^22,28^ The impact of altered βIII-tubulin levels has been noted in clinical and in vitro studies, which confirms the crucial role of elevated βIII-tubulin levels in the resistance of cancer cells to a broad spectrum of drugs.^29–32^ In addition, βIII-tubulin has been shown to be involved in tumor development and invasiveness.^33–36^ Therefore, novel compounds that exhibit similar potency but are less prone to drug resistance are the need of the hour.

We had previously reported that when the *N*-benzoyl moiety in paclitaxel is replaced with a ferrocenyl group, it enhances its cytotoxicity and the ability to promote tubulin polymerization.^37–39^ Moreover, simple substitution or conjugation of an organic molecule with the ferrocenyl group may considerably influence the mode of action of a given molecule and even lead to novel bioactivity. For example, when the phenyl group in plinabulin is replaced with the ferrocenyl moiety, a potent MDR protein inhibitor is obtained.^40^ In continuation of our efforts to assess organometallic antimitotic agents,^37,41–47^ the therapeutic potential of taxanes bearing bulky organometallic or organic groups instead of *N*-benzoyl moieties was investigated in this study by exploring the ferrocenyl, ruthenocenyl, and 1-adamantyl analogs of paclitaxel. The effects of these substituents on the biological properties of the tested taxanes were determined.

## Results and Discussion

Taxanes **1a–c** were synthesized using a three-step procedure starting from (*3R,4S*)-4-phenyl-3-(triethylsilyloxy)­azetidin-2-one **3** and 7-O-triethylsilylbaccatin III **5**, as previously described for **1a**.^39^ Initially, **3** was reacted with acyl chlorides **2a–c** in DCM in the presence of Et_3_N at room temperature (rt), yielding 52, 73, and 98% of **4a**, **4b** and **4c**, respectively. In the next step, **5** was reacted with LiHMDS and an excess of **4a–c** at −40 °C to obtain TES-protected taxanes **6a**, **6b**, and **6c** with 74, 33, and 60% yields, respectively. The obtained products were further reacted with an excess of hydrogen fluoride in pyridine at rt for 24 h, leading to the desired taxanes **1a**, **1b**, and **1c** with 90, 80, and 94% yields, respectively (Scheme 1).

**Scheme 1:**
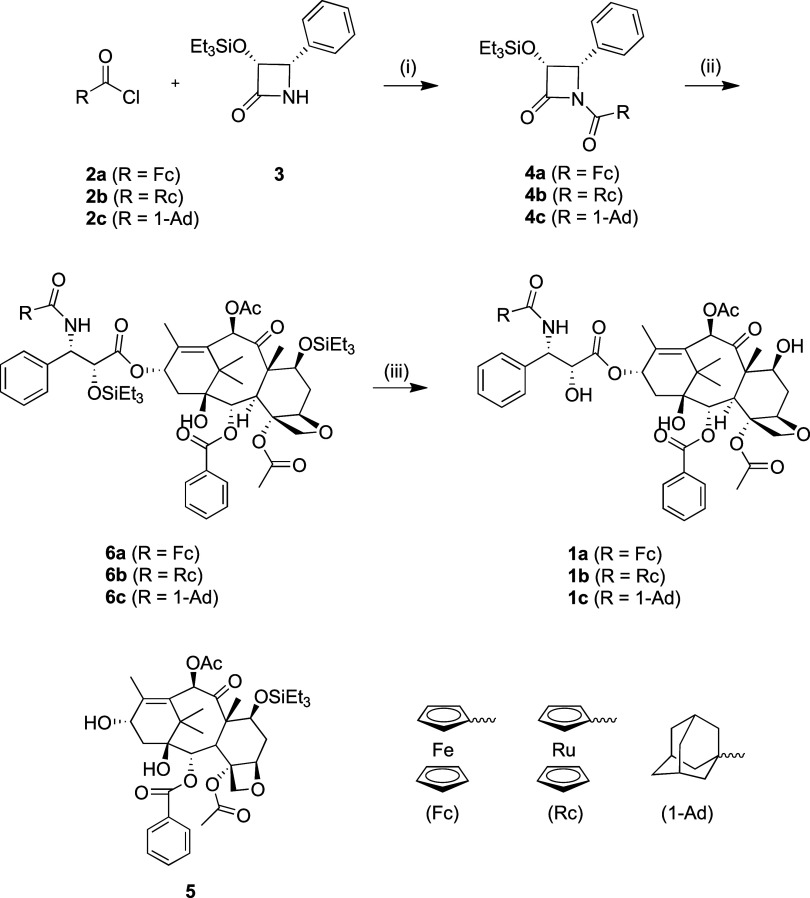
Synthesis of the Ferrocenoyl **1a**, Ruthenocenoyl **1b**, and 1-Adamantanoyl **1c** Analogs of Paclitaxel **1^a^**

a Reagents and conditions: (i) Et_3_N, DCM, rt, 52–98% (ii) LiHMDS, 5, THF, −40 °C, 40 min, 33–74%; (iii) HF, Py, rt, 24 h, 80–94%.

### New Taxane Analogs Exhibited Greater Cytotoxicity toward Colon Cancer Cells than Did Paclitaxel

Initially, the antiproliferative activity of the investigated compounds was screened against a noncancerous colon epithelial cell line CCD841 CoN, and a panel of colon adenocarcinoma cell lines (Table 1) isolated from different developmental stages, namely, LS180, HCT116, SW620, and LoVo. The cytotoxic/cytostatic effects depend strongly on the *N*-acyl substituent. The ferrocenyl analogue of paclitaxel **1a** was similar to that of **1** with IC_50_ values in the micromolar range, whereas ruthenocenyl **1b** and 1-adamantyl **1c** derivatives displayed substantially greater activity with IC_50_ values in the low submicromolar or nanomolar range (Table 1). Furthermore, **1c** did not demonstrate any difference in activity among the studied colorectal cancer cell lines; on the contrary, **1a** and **1b** were the most active against LoVo cells. Importantly, all tested compounds exhibited significantly lower activity toward the noncancerous colon epithelial cell line CCD841 CoN, indicating a certain degree of selectivity for cancer cells.

**Table 1:** Cytotoxicity of Paclitaxel **1** and Its Analogs **1a–c** in a Noncancerous Colon Epithelial Cell Line and Colorectal Cancer Cell Lines^a^

compound	CCD841 CoN	LS180	SW620	HCT116	LoVo
**1**	314 (139–2848)	1.12 (0.799–1.66)	1.02 (0.690–1.64)	8.14 (1.61–23.8)	1.02 (0.786–1.36)
**1a**	235 (163–1400)	4.58 (3.40–6.74)	1.82 (1.45–2.35)	2.95 (2.06–4.81)	1.72 (1.55–1.91)
**1b**	53.1 (37.6–83.9)	0.279 (0.149–0.711)	0.102 (0.065–0.162)	0.132 (0.069–0.319)	0.045 (0.028–0.066)
**1c**	25.9 (14.8–76.6)	0.074 (0.057–0.094)	0.075 (0.057–0.098)	0.085 (0.075–0.094)	0.086 (0.061–0.123)

a The results are presented as IC_50_ values [μM] calculated from three independent experiments and their corresponding 95% confidence intervals (in parentheses).

Next, the most active compounds **1b** and **1c** were tested by using a three-dimensional model derived from LoVo cells. The findings indicated that **1** and **1b**–**c** at concentrations equal to the IC_50_ markedly reduced spheroid growth within 8 days to a virtually equal extent (Figure 1).

**Figure 1:**
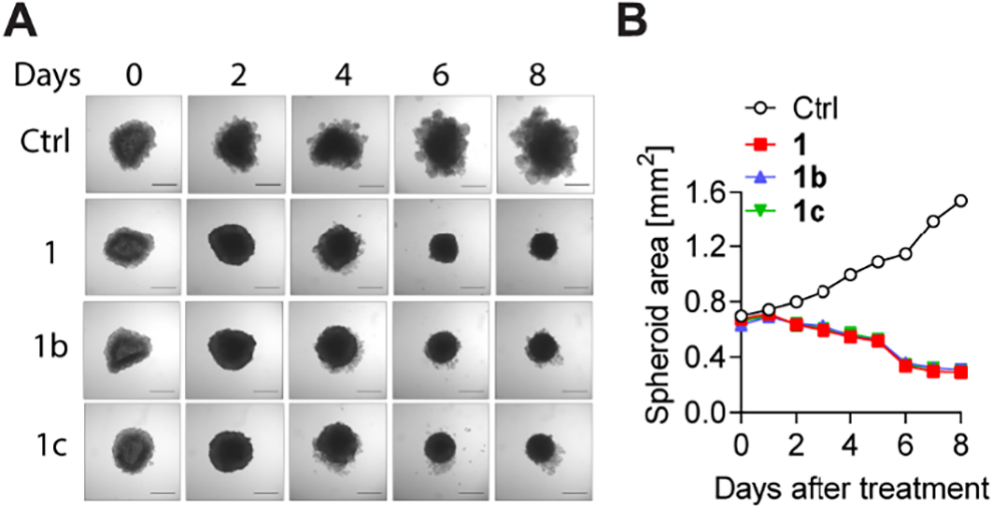
LoVo cell spheroid cultures treated with **1** or its analogs **1b–c**. (A) Representative bright-field optical microscopy images of spheroids cultured in the absence or presence of **1**, **1b**, or **1c** on day 0, day 2, day 4, day 6, and day 8. (B) Time-course variations in the focal plane area of spheroids treated with the investigated compounds. The area (mm^2^) was presented as the mean ± SD (*n* = 3). Scale bar, 500 μm.

A typical feature of taxane-exposed cells is cell cycle arrest at the metaphase-anaphase junction, which results from the stabilization of microtubules and reduces the critical concentration of free tubulin. Accordingly, the cell cycle phase distribution in colorectal cancer cells was investigated in the presence of **1** or **1a–c** for 12, 24, and 48 h. In all taxane-treated cells, the number of M-phase arrested cells and the number of apoptotic events, as evidenced by the so-called “sub-G1” fraction, increased over time (Figure S1). Nonetheless, the dynamics of the process were markedly more rapid in cells incubated with metallocenyl paclitaxel analogs **1a** and **1b**, as effects were observed after 24 h of exposure itself. These results testified that the cytotoxic effects were more pronounced in cells exposed to ferrocenyl and ruthenocenyl taxane analogs.

### New Taxane Analogs Exerted a Slight Effect on Reactive Oxygen Species Production

The biological activity of taxanes can, at least partially, be attributed to their effects on the intracellular redox balance. Paclitaxel significantly increased the generation of reactive oxygen species (ROS) in ovcar-3 cells by interacting with complex IV (cytochrome c oxidase).^48^ Paclitaxel activates plasma membrane-bound NADP oxidase, which leads to the excessive generation of H_2_O_2_ and the bystander toxicity phenomenon observed in cell culture conditions.^49^ These effects appear to result from the interactions of tubulin-bound paclitaxel with enzymes rather than the pro-oxidative properties of taxanes, as other tubulin-binding agents also produced a similar outcome. However, it appears that organometallic anticancer compounds, particularly ferrocene derivatives, can directly generate ROS via the Fenton-like reaction, which might lead to improved cytotoxicity.^50–52^ Therefore, cell exposure to organometallic taxane analogs can be assumed to result in elevated ROS production. To examine the pro-oxidative potential of the studied compounds, a dihydrorhodamine 123 (DHR123) oxidation assay was employed (Figure S2). In all cases, the intracellular ROS level was elevated in taxane-exposed cells compared to control cells, although the magnitude of the effect varied between compounds and cell lines. The ferrocenyl analog **1a** exhibited more robust pro-oxidative properties than its purely organic counterparts **1** and **1c** in LS180 and SW620 but not in LoVo cells. The effects of ruthenocenyl **1b** were comparable to those of paclitaxel although slightly higher than those of **1c**. The least variability among different taxanes was observed in the SW620 cells. The findings did not show a clear correlation between ROS generation and the antiproliferative potency of the investigated compounds, which implies that this mechanism is not of major significance for the mode of action of the analogs.

### Effects of Taxane Analogs on Microtubule Subunit Composition

Based on the knowledge that **1** can stabilize microtubules,^8^ the effects of paclitaxel analogs **1a–c** on the rate of microtubule polymerization were examined (Figure 2). An association was observed between the stage of cancer development and the microtubule polymerization rate in cells treated with **1b** or **1c**. In contrast, in the **1a**-treated cells, changes were not observed in microtubule polymerization compared with **1**-treated cells. The most significant effect on the polymerization rate was seen for microtubules isolated from LoVo cells, which displayed a notably decreased polymerization rate in the **1b**-treated cells and a slight change in **1c**-treated cells compared with **1**-treated cells. To quantify the polymerization dynamics shown in Figure 2, we extracted the initial slope (i.e., the angle of the steepest portion of the curve), which reflects the rate of microtubule polymerization for each condition (Figure S3). This graphical analysis reveals that compound **1b** induces a noticeably lower polymerization rate in HCT116 and LoVo cells compared to compounds **1** and **1a**. These observations are consistent with the delayed polymer growth and lower plateau levels seen in Figure 2.

**Figure 2:**
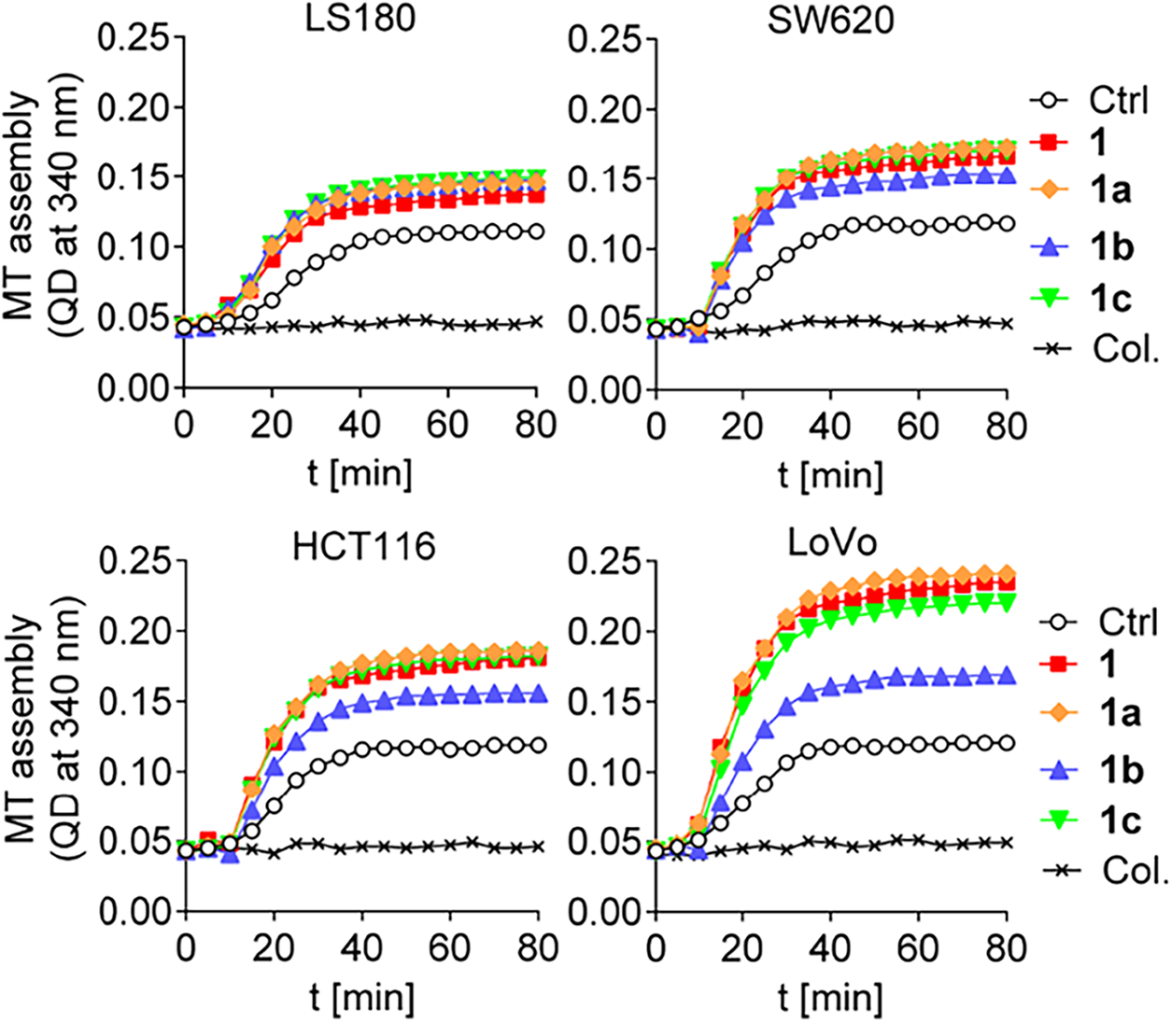
Polymerization of tubulin (10 mg/mL) isolated from LS180, SW620, HCT116, and LoVo cells treated previously with **1**, colchicine (Col.), and **1a–c** at a 3 μM concentration of compounds. Results of a single representative experiment are presented.

The observed alterations in cytotoxicity and the rate of microtubule polymerization could be ascribed to the modulation of tubulin composition.^20^ Altered β-tubulin isotypes play a prominent role in the anticancer activity of taxane.^19^ βIII-tubulin is upregulated in advanced cancers.^53–55^ A high level of this tubulin is correlated with a more rapid microtubule polymerization rate and, consequently, accelerated cellular movement.^23^ Conversely, when βIII-tubulin is silenced, it leads to slower cancer cell migration and reduced invasiveness.^36^ Moreover, high βIII-tubulin expression is linked to aggressive tumor behavior, as is evidenced in colorectal, breast, ovarian, prostate, and pancreatic cancers.^26,56–59^ A cellular model for studying tumor aggressiveness is the induction of epithelial-to-mesenchymal transition (EMT) in cells.^60^ In colorectal cancer, βIII-tubulin is abundantly expressed in tumor buds, which are small clusters of dedifferentiated tumor cells undergoing EMT and located at the invasive front,^35^ which is the area where the most aggressive cancer cells are present. These cells are known for their ability to invade surrounding tissues, acquire new traits, resist various forms of therapy, and suppress the immune response. This region is of paramount importance for cancer progression, as it represents the forefront of tumor expansion and metastasis.

By contrast, βIVa-tubulin expression exerted opposite effects on cell motility. Increased levels of this tubulin reduced cell migration, whereas its downregulation stimulated the movement of metastatic cancer cells.^61^ Considering these findings, the impact of the taxane analogs on the expression of different β-tubulin isoforms (encoded by *TUBB1*, *TUBB2*, *TUBB3*, *TUBB4B*, and *TUBB6*) was studied. The cells were incubated with **1** and **1a–c** (at concentrations equal to the IC_90_ of **1** for the respective cell lines), and the levels of different β-tubulin isoforms in the isolated microtubule fractions were determined by using Western blotting. The downregulation of βIII-tubulin expression (2- to 12-fold) was strictly accompanied by the upregulation of βIVa-tubulin expression (up to 2-fold) in SW620, HCT116, and LoVo cells exposed to **1b** or **1c** (Figure 3). The effects of the ruthenocenyl analogue **1b** were more pronounced than those of its adamantyl counterpart **1c**. Neither **1** nor **1a** exerted a similar effect. None of the investigated compounds influenced the βIII-tubulin/βIVa-tubulin levels in LS180 cells. Moreover, none of the *N*-acyl-substituted taxanes affected the expression levels of βI-tubulin, βII-tubulin, or βVI-tubulin (Figure S4).

**Figure 3:**
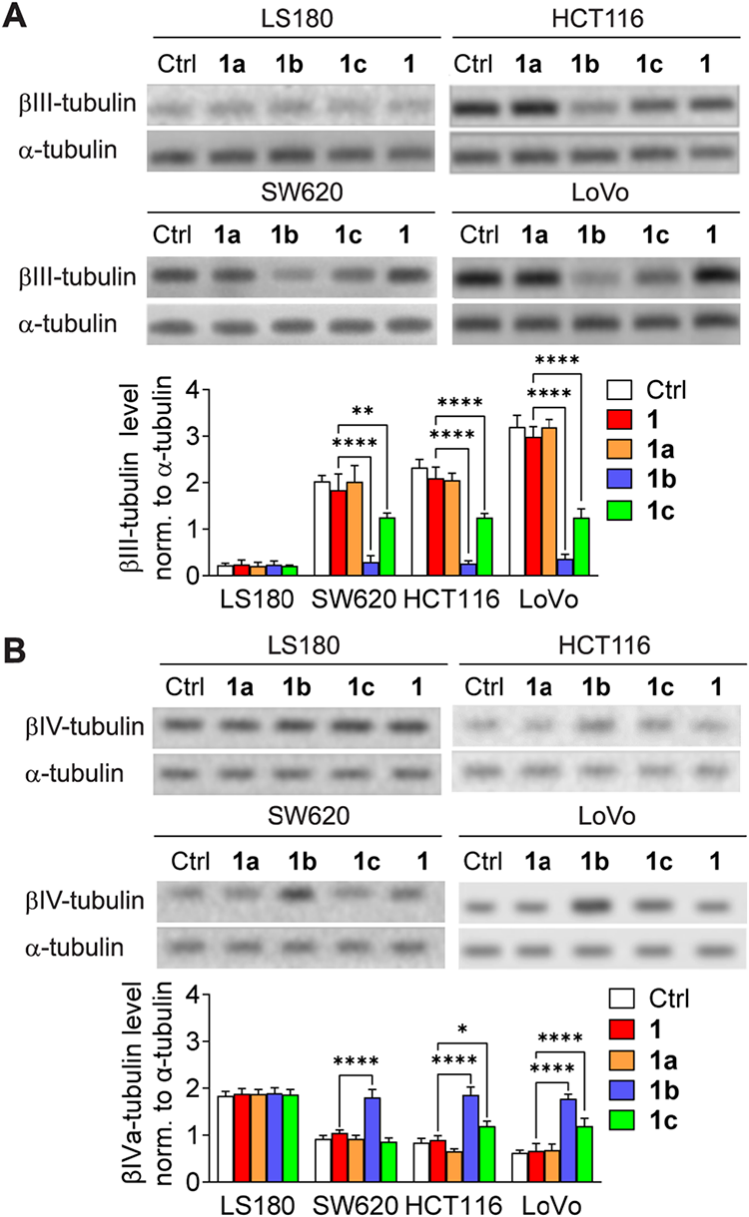
Modulation of (A) βIII-tubulin and (B) βIVa-tubulin protein levels in LS180, SW620, HCT116, and LoVo cells treated with **1** or **1a–c**. Representative Western blot results are shown. Graphs indicate the mean optical density of βIII-tubulin or βIVa-tubulin normalized to corresponding TUBA bands ± SD (*n* = 3), *p < 0.05, **p < 0.01, ****p < 0.001.

Interestingly, the distinct responses to **1b** and **1c** in LS180 versus LoVo cells may be attributed to differences in the expression of tubulin isotypes in these cell lines. LoVo cells are characterized by elevated βIII-tubulin levels, which have been associated with increased microtubule dynamics and reduced sensitivity to taxanes.^19^ In contrast, LS180 cells express lower levels of βIII-tubulin, which may explain the lack of compound-specific variation in polymerization. As shown in Figure 3, compounds **1b** and **1c** significantly downregulate βIII-tubulin and upregulate βIVa-tubulin in LoVo cells, whereas no such modulation was observed in LS180. This suggests that the observed gradation in tubulin polymerization rates in LoVo may be mechanistically linked to the ability of the compounds to modulate specific β-tubulin isotypes.

### Taxane Analogs Inhibited the Invasion Ability of Colon Cancer Cells via β-Tubulin Modulation

As taxane analogs modulate β-tubulin subunits and the most substantial effect was perceived in cells with an invasive phenotype, whether these analogs affect cell invasiveness was examined. The cells were incubated with each studied compound at a concentration equal to the IC_90_ value of **1** for the respective cell lines. In **1b**-treated cells, invasion was inhibited by 27, 28, and 75% concentrations in SW620, HCT116, and LoVo cells, respectively, compared with nontreated cells (Figure 4A).

**Figure 4:**
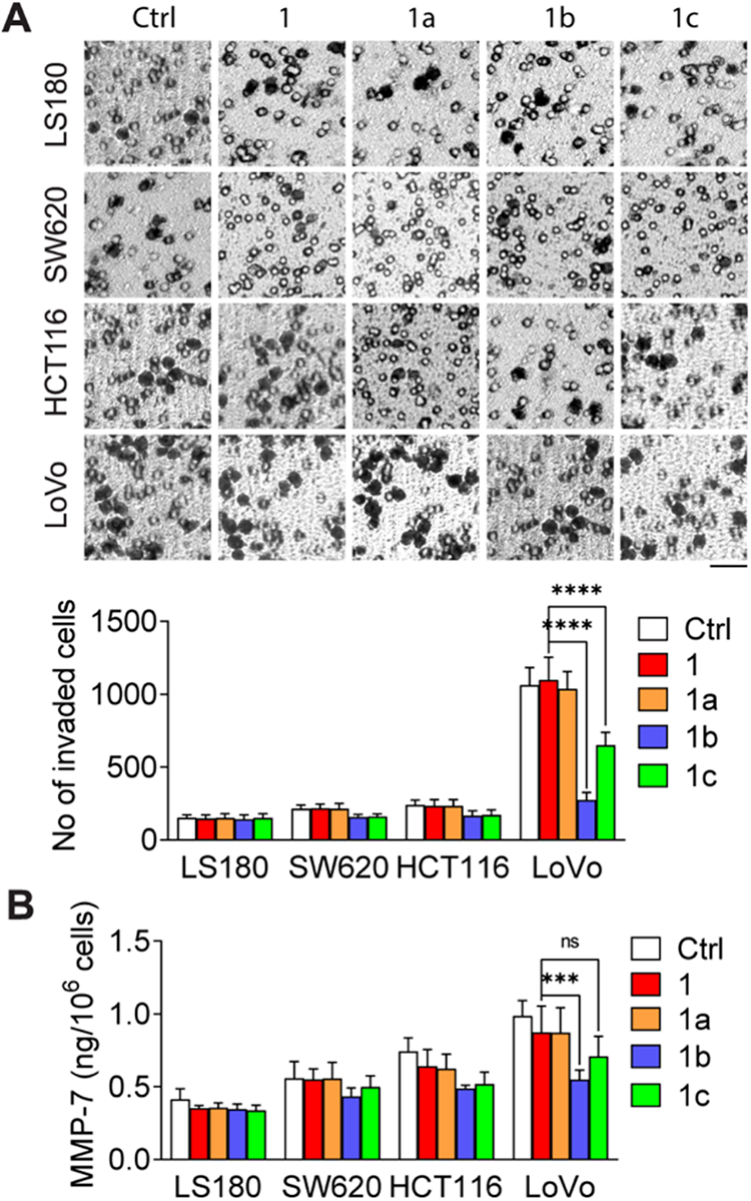
(A) Representative images and quantification of invaded cells in LS180, SW620, HCT116, and LoVo cell lines after 8 h of treatment with **1**, **1a**, **1b**, or **1c** (3 μM). Invasion was assessed using Matrigel-coated polycarbonate membrane inserts with 8.0 μm pores. The number of invaded cells was counted in five random microscopic fields per condition. A clear and statistically significant reduction in cell invasion was observed only in LoVo cells treated with **1b** and **1c** compared to control and paclitaxel **1** (****p < 0.0001). In the remaining cell lines (LS180, SW620, HCT116), treatment effects were limited and not statistically significant, although small nonsignificant trends were observed. (B) Secretion levels of MMP-7 measured in culture media collected from treated cells after 48 h, as determined by ELISA. A significant decrease in MMP-7 secretion was observed only in LoVo cells treated with **1b** and **1c** (***p < 0.001). Data represents mean ± SD from three independent experiments. ns—not significant.

A weak inhibitory effect was observed for **1c**, whereas **1** and **1a** did not affect the cells. Also, none of the tested compounds showed anti-invasive effects on LS180 cells. The effects of taxane analogs on the secretion of matrix metalloproteinase-7 (MMP-7), a zinc endopeptidase that digests extracellular matrix components, were subsequently studied. Microtubules facilitate the trafficking of MMP-7 to invadopodia—structures essential for the invasiveness of cancer cells in advanced tumor stages.^28^ Compared with no treatment, incubation of SW620, HCT116, and LoVo cells with **1b** reduced MMP-7 secretion by 20, 35, and 45%, respectively (Figure 4B). While slightly lower effects were demonstrated for **1c**, **1** and **1a** did not modulate MMP-7 secretion. Hence, decreased MMP-7 secretion, which is strongly correlated with the results of cellular motility, appears to be linked to altered polymerization of microtubules in cells treated with **1b** and **1c**.

To confirm that taxane analogs exerted greater effects on cells with an invasive potential, their effects on cell lines isolated from patients with advanced colorectal cancer who did not receive chemotherapy were determined. Previously established cell lines were categorized into two cohorts: low grade (cells isolated from patients with stage I and II disease) and high grade (cells isolated from patients with stage III and IV disease; Figure 5A and Table S1). The results for cells isolated from patients with colon cancer were consistent with those obtained from a panel of colorectal cancer cell lines (LS180, SW620, HCT116, and LoVo; Figure 5B,C).

**Figure 5:**
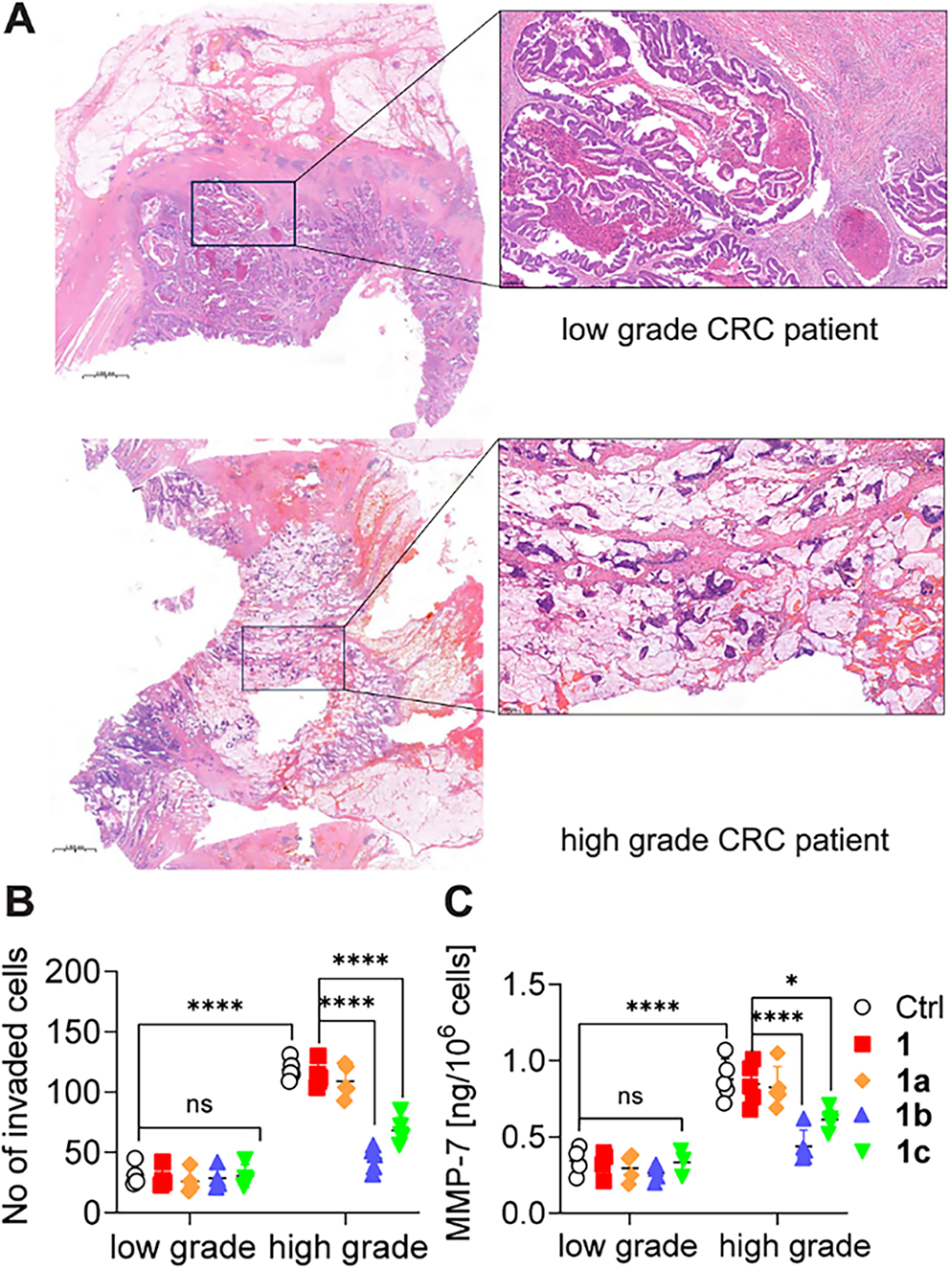
Effects of **1** and its analogs **1a–c** on the invasion abilities of patient-derived colon cancer cells. (A) Images of carcinoma cells in the colon cancer tissue stained with hematoxylin and eosin (scale bar 2 mm), (B) invasion abilities, and (C) MMP-7 secretions of cancer cells treated with **1** and its analogs **1a–c**. Each dot in (B) and (C) represents a unique, individual primary culture derived from patients with either low-grade or high-grade colorectal cancer (*n* = 4). Representative images are shown; the graphs display mean ± SD (*n* = 4), *p < 0.05, ****p < 0.001.

Next, whether the effects of inhibiting invasion were due to the modulation of β-tubulin subunit levels in cells treated with taxane derivatives was investigated. Microtubules rich in βIII-tubulin and poor in βIVa-tubulin were hypothesized to play a critical role in the regulation of **1b**- or **1c**-treated colon cancer cell invasiveness.

Thus, the decision was made to modulate the levels of the tubulin subunits. Initially, the effects of taxane analogs in βIII-tubulin-overexpressing colon cancer cells were studied. Increasing the βIII-tubulin level by 1.6-fold resulted in decreased inhibition of invasion in the **1b**- or **1c**-treated cells (Figure 6A,B). The potency of invasion inhibition by **1b** or **1c** was reduced by 30% or 24%, respectively, in LoVo cells overexpressing βIII-tubulin. Subsequently, the effects of the taxane analogs were studied in βIVa-tubulin-silenced cells, which revealed that the downregulation of βIVa-tubulin by 90% decreased the invasion of cells treated with **1b** or **1c** by 47 or 33%, respectively (Figure 6C,D). These observations confirmed that **1b–c** taxane analogs inhibited colon cancer invasion by regulating the βIII-tubulin/βIVa-tubulin subunit balance within microtubules. βIII-tubulin is often upregulated during tumor progression and is associated with increased aggressiveness and resistance to therapy. While paclitaxel effectively stabilizes microtubules, it does not reverse the βIII-tubulin-associated signaling changes or prevent its overexpression. In contrast, compounds **1b** and **1c** not only reduce βIII expression but also upregulate βIVa, suggesting a distinct mechanism that may underlie their anti-invasive effects.

**Figure 6:**
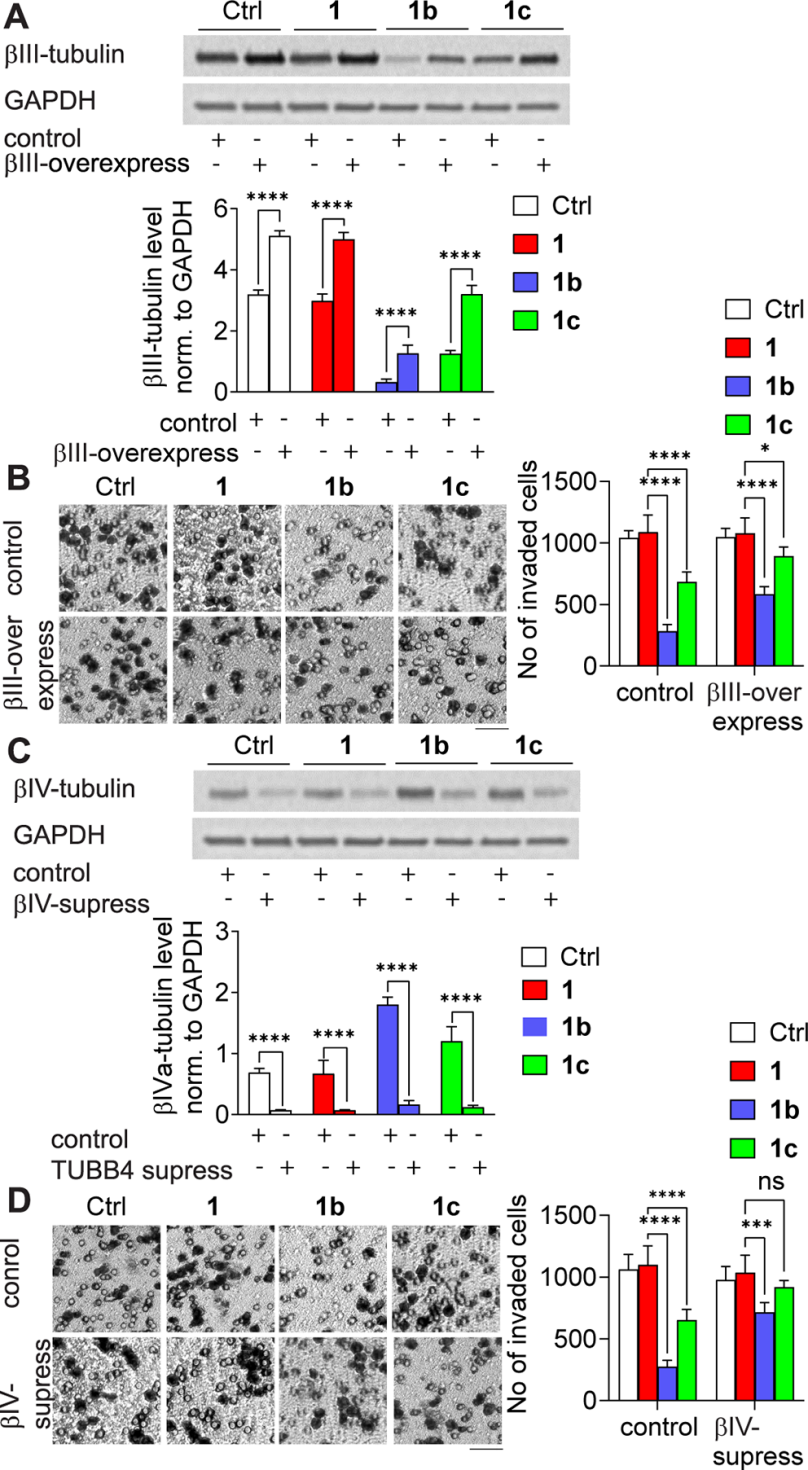
Modulation of invasion abilities in βIII-tubulin-overexpressed or βIVa-tubulin-silenced LoVo cells treated with **1** or its analogs **1b–c**. The level of (A) βIII-tubulin in βIII-overexpressed cells and (C) βIVa-tubulin in βIVa-suppressed cells. Representative Western blot results are shown, with the graphs presenting the mean optical density of βIII-tubulin or βIVa-tubulin normalized to GAPDH ± SD (*n* = 3), ****p < 0.001. Invasion abilities of (B) βIII-overexpressed cells and (D) βIVa-suppressed cells (scale bar = 100 μm). Representative images are shown (the ring is a pore in the membrane, and the black dot is a cell); the graphs display mean ± SD (*n* = 3), *p < 0.05, ***p < 0.005, ****p < 0.001.

### Taxane Analogs Affected Focal Adhesion Sites via Modulation of the Microtubule–Integrin-Linked Kinase–Integrin−β1 Axis

Alterations in the TUBB isoform composition in cancer cells may influence integrin-linked kinase (ILK) stabilization in the vicinity of the plasma membrane and affect the organization of focal adhesion sites (FAs). Western blot analysis of ILK levels in the cytoplasmic fraction of **1b-** or **1c**-treated cells indicated a reduction in these levels (Figure 7), whereas the total protein level remained unchanged (Figure S5). The most significant alterations were observed with **1c** treatment (approximately 70% reduction compared with control cells), whereas **1b** exposure resulted in >30% decrease in the cytosolic concentration of ILK. Accordingly, either βIII-tubulin overexpression or βIVa-tubulin silencing significantly restored the ILK level (Figure 7). Collectively, these findings indicate that increased tumor invasiveness is correlated with ILK upregulation, as reported elsewhere.^62^

**Figure 7:**
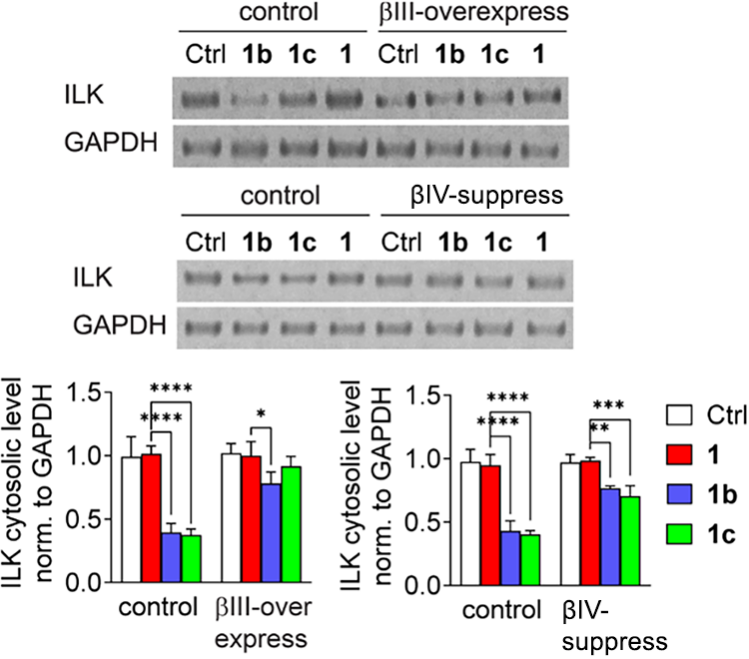
Modulation of cytosolic ILK in βIII-tubulin-overexpressed or βIVa-suppressed LoVo cells treated with **1** or its analogs **1b–c**. Representative Western blot results are shown, with the graphs showing the mean optical density of cytosolic ILK normalized to GAPDH ± SD (*n* = 3), *p < 0.05, **p < 0.01, ***p < 0.005, ****p < 0.001.

As reported by numerous studies, ILK activity is linked to the conformation of β1-integrins, integrin clustering, and FA maturation.^63^ The ILK E359 K mutation has previously been shown to result in a decreased number of β1 integrin clusters.^64^ In addition, ILK silencing reduces migration and invasion via the disruption of integrin localization or cluster formation in the plasma membrane of advanced tumor cells.^65^ Reduced invasiveness manifests as a decrease in the plasma membrane pool of integrin-β1, which leads to a decrease in the FA number. Therefore, whether the cytosolic level of ILK affects integrin-β1 intracellular localization and FA organization^66^ was investigated. The results signified that compared with **1**-treated cells, LoVo cells treated with **1b** and **1c** showed a 2-fold decrease in the amount of integrin-β1 in the plasma membrane (Figure 8A).

**Figure 8:**
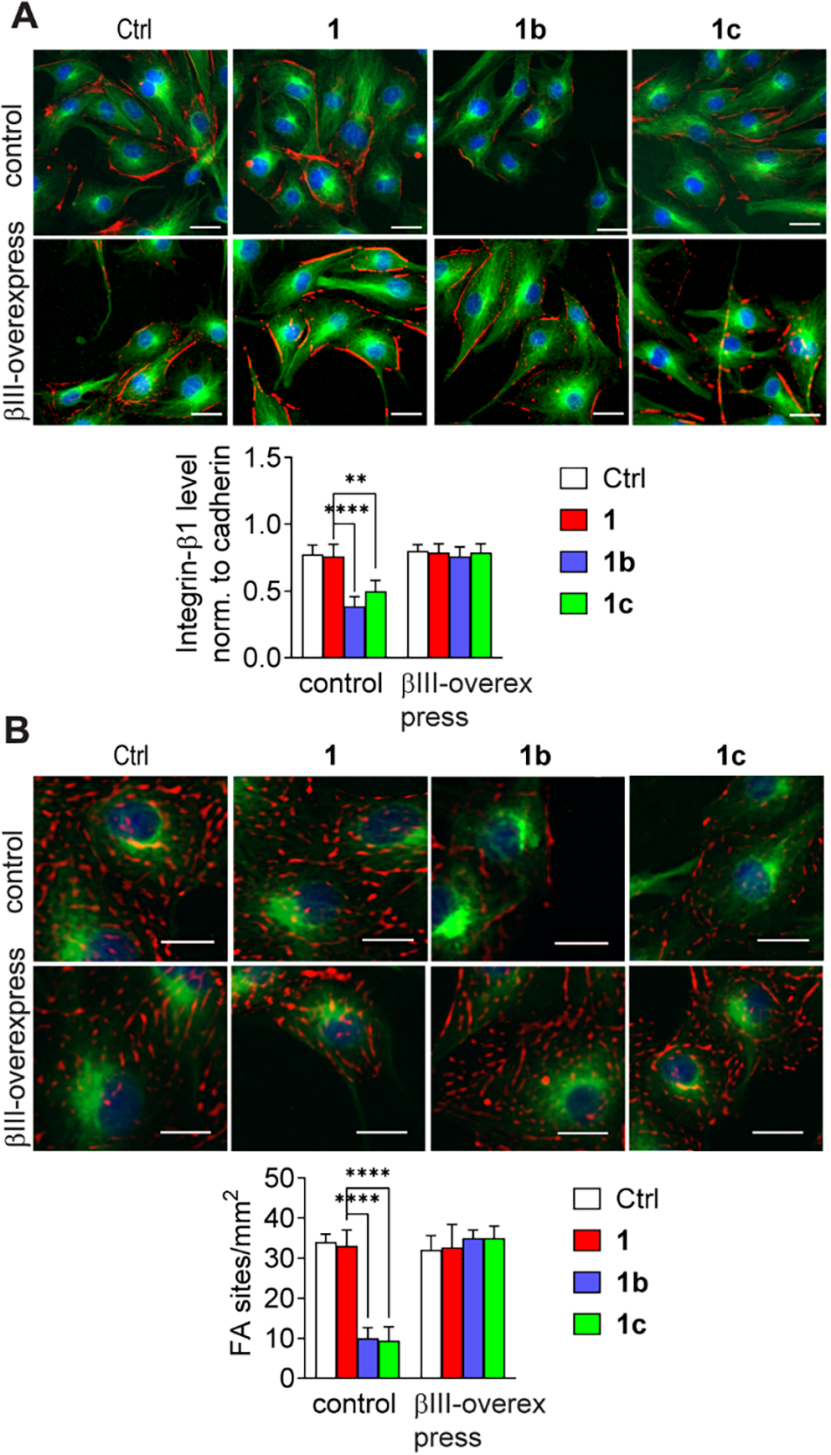
Analysis of focal adhesion site elements in LoVo cells exposed to **1** or its analogs **1b–c**. (A) Representative fluorescence microscopic images of cells showing the localization of integrin-β1 (red), cadherins (green), and the nucleus (blue). The graph presents the mean fluorescence intensity of integrin-β1 normalized to cadherin ± SD (*n* = 3), **p < 0.01, ****p < 0.001. Scale bar 50 μm. (B) Representative fluorescence microscopic images of cells showing FA localization (labeled using TRITC-conjugated antivinculin antibodies), microtubules (green), and nucleus (blue). The graph presents the mean of FA number sites per mm^2^ ± SD (*n* = 3), ****p < 0.001. Scale bar 50 μm.

Interestingly, the findings revealed that βIII-tubulin overexpression induced these effects. Furthermore, an approximately 50% decrease in the number of FAs was observed in the **1b-** and **1c**-treated cells (Figure 8B). From these results, it can be concluded that **1b/1c** treatment alters the expression of tubulin subunits by downregulating βIII-tubulin while enhancing the upregulation of βIVa-tubulin. ILK activity decreases, which results in a reduction in the pool of integrin-β1 in the cell membrane as well as the number of FA sites.

### New Taxane Analogs Did Not Induce Chemoresistance via βIII-Tubulin Overexpression

Long-term administration of paclitaxel has been associated with βIII-tubulin overexpression and the development of taxane resistance both in vitro and in vivo. Poor efficiency of taxane-based treatment has been reported in non-small-cell lung cancer, uterine serous carcinoma, advanced gastric cancer, breast cancer, and ovarian cancer when *TUBB3* is overexpressed.^67^ The mechanism of resistance was explained by studies on molecular dynamics, which revealed the reduced paclitaxel affinity of microtubules enriched in βIII-tubulin subunits.^19^ Accordingly, unlike paclitaxel, which does not appear to inhibit βIII-tubulin overexpression, compounds **1b** and **1c** may actively prevent the development of βIII-tubulin-dependent resistance. To test this hypothesis, we investigated whether prolonged **1b** or **1c** exposure could lead to resistance development was investigated. The total βIII-tubulin and βIVa-tubulin levels were determined in cells cultured in the presence of **1a–c** or **1** for 3 days (Figure S6) or 6 weeks (the medium was replaced every 84 h with a new portion of the investigated agents in fresh medium; Figure 9). The concentration of the investigated taxanes was equal to the IC_90_ value for paclitaxel in a specific cell line. As expected, 3 days of treatment did not affect either protein (Figure S6), but long-term incubation in the presence of **1c** altered the expression levels of both βIII-tubulin and βIVa-tubulin in HCT116 and LoVo cell lines. The effects of the **1b** treatment were also explored in SW620 cells. Long-term treatment of cells with **1b** or **1c** led to decreased βIII-tubulin levels (Figure 9A) and increased βIVa-tubulin levels (Figure 9B). These intriguing observations contradict the aforementioned literature reports. It may be speculated that ruthenocenyl and adamantyl paclitaxel analogs specifically modulate the expression of TUBB isoforms, thus promoting βIVa-tubulin biosynthesis; however, the underlying mechanism is yet to be elucidated.

**Figure 9:**
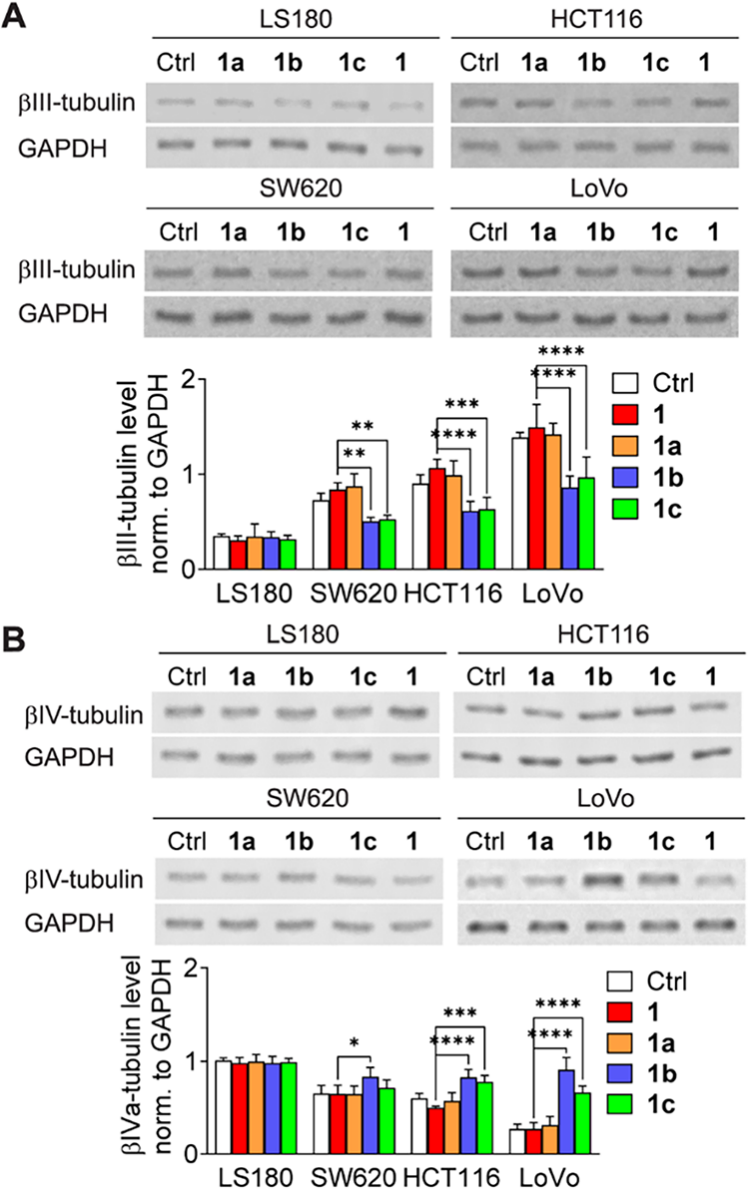
Alterations in total (A) βIII-tubulin and (B) βIVa-tubulin levels in cells chronically treated with **1** or its analogs **1a–c**. Representative Western blot results are shown, with the graphs presenting the mean optical density of βIII-tubulin or βIVa-tubulin normalized to GAPDH ± SD (*n* = 3), *p < 0.05, **p < 0.01, ***p < 0.005, ****p < 0.001.

### New Taxane Analogs Are Still Recognizable by ABCB1

Modification of the molecular structure may considerably alter the pharmacokinetic profile of a given compound, considering recognition by different transport systems that form intrasystemic barriers (e.g., gut–blood or blood–brain barrier). On the contrary, only agents that achieve an adequate intracellular concentration can exert biological effects. Therefore, whether modifications of the core taxane structure influenced the recognition of novel molecules by major membrane transport systems was investigated. As paclitaxel and docetaxel are known substrates of ABCB1 (also known as P-gp, an MDR protein that is a major constituent of blood–organ barriers),^68^ an assay that utilized a UIC2 antibody that differentiates the allocrite-bound conformation of the ABCB1 protein molecule was performed. The level of antibody binding was comparable for all investigated taxanes, although it was approximately 20% lower than that for the positive control (vincristine; Figure S7). These findings imply that the *N*-acyl substituent of paclitaxel does not influence the ability of ABCB1 to recognize taxane molecules.

## Conclusions

In summary, the novel paclitaxel analogs **1b** and **1c** exhibited at least 10 times greater cytotoxicity toward colon cancer cells than did paclitaxel. Furthermore, paclitaxel analogs exhibited a potency to modulate the invasiveness of colon cancer cells via regulation of tubulin subunits. The presence of βIII-tubulin in microtubules made their structures more dynamic and enhanced their resistance to taxanes. Importantly, novel paclitaxel analogs **1b** and **1c** significantly reduced βIII-tubulin levels, leading to markedly slower microtubule polymerization and a consequent reduction in the inclusiveness of colon cancer cells. βIII-tubulin is a protein critically involved in cancer metastasis and is known to be present in invading cell bubbles during colon cancer development. In addition, a simultaneous increase in βIV-tubulin levels was observed, which was associated with a reduced cell motility and invasiveness. This dual modulation of βIII- and βIV-tubulins was responsible for the unique capability of these compounds to alter microtubule dynamics and inhibit cancer cell migration and invasiveness. Furthermore, the disruption of FA organization via the mislocalization of ILK and the consequent interruption of the integrin signaling pathways underscored the multifaceted effects of these compounds. Notably, prolonged exposure to **1b** or **1c** not only avoided triggering paclitaxel-associated chemoresistance pathways but also actively promoted antiresistance mechanisms. These novel findings indicate that the ruthenocenoyl and 1-adamantanoyl analogs of paclitaxel could be useful in the development of novel therapies that simultaneously target cancer growth and metastasis, offering a promising avenue for effective in situ cancer treatment and metastasis prevention.

## Experimental Section

### General

^1^H and ^13^C­{^1^H} NMR spectra were recorded on a Bruker ARX 600 MHz instrument (600.26 MHz for ^1^H and 150.95 MHz for ^13^C). Chemical shifts in ^1^H and ^13^C NMR spectra are referenced relative to residual solvent signals: CDCl_3_ 7.26 ppm for ^1^H and 77.0 ppm for ^13^C, DMSO-*d*_6_ 2.50 ppm for ^1^H and 39.5 ppm for ^13^C. Spectra were recorded at room temperature, 293 K; chemical shifts are in ppm, and coupling constants in Hz. Thin-layer chromatography (TLC) was performed on aluminum sheets precoated with Merck 5735 Kieselgel 60 F254. Column chromatography was carried out on Fluka Silica gel 60 for flash chromatography (0.040–0.063 mm, 230–400 mesh). All reactions were carried out using the standard Schlenk technique. Chemicals, biochemicals, and solvents (HPLC grade) were purchased from Sigma-Aldrich, AK Scientific or HBCChem, Inc. and used as received unless stated otherwise. Dichloromethane was distilled from calcium hydride and stored over activated molecular sieves 4Å (8–12 mesh). Acetonitrile was distilled from P_4_O_10_. The purity of final compounds was ≥95% as measured by HPLC and elemental analysis. The HPLC-MS analysis was performed on an analytical Phenomenex Kinetex XB-C18 column (50 × 4.6 mm, 1.7 μm) using a Shimadzu Nexera XR system equipped with SPD-M40 and LCMS-2020 detectors. The mobile phase consisted of solvent A (water +0.01% HCOOH) and solvent B (methanol +0.01% HCOOH). A gradient elution was applied as follows: 30% of B at 0 min, increased linearly to 90% of B at 5 min, held for 1 min (until 6 min), then returned to 30% of B at 8 min, and held until 15 min. The flow rate was set at 0.3 mL·min^–1^. Compounds **1a**, **4a**, and **6a** were synthesized as described previously.^39^ The HPLC analysis confirmed a purity of >95% for **1a**–**c**.

### Synthesis

#### Ruthenocenecarbonyl Chloride **2b**

505 mg (340 μL, 4.0 mmol) of oxalyl chloride followed by 1 drop of DMF were added to a slurry of 500 mg (2.0 mmol) ruthenocenecarboxylic acid in 10 mL of anhydrous dichloromethane. The resulting mixture was stirred at RT for 1 h and evaporated to dryness. The crude product was dried under vacuum for 30 min and used for the next step without further purification.

#### (*3R,4S*)-3-(Triethylsilyloxy)-4-phenyl-1-ruthenocenoyl-azetidin-2-one **4b**

The solution of freshly prepared ruthenocenecarbonyl chloride **2b** in 5 mL of anhydrous dichloromethane was added dropwise to a solution of 470 mg (1.7 mmol) of (*3R,4S*)-4-phenyl-3-(triethylsilyloxy)­azetidin-2-one **3**, 206 mg (1.7 mmol) DMAP, and 607 mg (836 μL, 6.0 mmol) of anhydrous triethylamine in 5 mL of anhydrous dichloromethane, and the reaction mixture was stirred at RT for 2 h. Next, the reaction was quenched by adding 50 mL of sodium bicarbonate, and the product was extracted with ethyl acetate (5 × 50 mL). The organic phases were combined, washed with brine, dried over sodium sulfate, and evaporated. Column chromatography on silica (150 g) using *n*-hexane-ethyl acetate (4–1) as an eluent gave 660 mg (73% yield) of pure **4b** isolated as pale-yellow oil. ^1^H NMR (CDCl_3_) δ 7.36–7.34 (m, 2H), 7.31–7.29 (m, 3H), 5.79–5.78 (m, 1H), 5.46–5.45 (m, 1H), 5.24 (d, *J* = 6.0 Hz, 1H), 5.03 (d, *J* = 6.0 Hz, 1H), 4.81–4.80 (m, 1H), 4.79–4.78 (m, 1H), 4.54 (s, 5H), 0.80 (t, *J* = 8.0 Hz, 9H), 0.53–0.42 (m, 6H); ^13^C­{^1^H} NMR (CDCl_3_) δ 167.3, 164.2, 134.2, 128.2, 120.1, 128.0, 75.1, 73.5, 73.1, 72.2, 72.1 60.6, 6.2, 4.5. Elemental analysis calcd for C_26_H_31_NO_3_RuSi C 58.40, H 5.84, N 2.62 found C 58.41, H 6.00, N 2.79.

#### (*3R,4S*)-1-(Adamatan-1-oyl)-3-(triethylosilyloxy)-4-phenylazetidin-2-one **4c**

This compound was synthesized in 98% yield (750 mg of pale-yellow oil) by the same procedure as described for **4b**, starting from 1-adamantanecarbonyl chloride **2c** instead of **2b**. ^1^H NMR (CDCl_3_) δ 7.32–7.30 (m, 2H); 7.28 (d, *J* = 7.1 Hz, 1H); 7.22–7.20 (m, 2H); 5.12 (d, *J* = 6.1 Hz, 1H); 4.99 (d, *J* = 6.1 Hz, 1H); 2.08–2.02 (m, 9H); 1.79–1.71 (m, 6H); 0.78 (t, *J* = 8.0 Hz, 9H); 0.49–0.41 (m, 6H); ^13^C­{^1^H} NMR (CDCl_3_) δ 176.5, 164.4, 134.3, 128.0, 127.7, 74.3, 60.5, 41.7, 36.4, 35.9, 27.9, 6.2, 4.4. Elemental analysis calcd for C_26_H_37_NO_3_Si C: 71.03, H 8.48, N 3.19 found C 71.01, H 8.69, N 2.91.

#### *N*-Debenzoyl-2′,7-O-bis­(triethylsilyl)-*N*-ruthenocenoylpaclitaxel **6b**

1.35 mL of 1.0 M LiHMDS in THF (1.35 mmol) was added to a solution of 458 mg (0.857 mmol) of **4b** and 400 mg (0.571 mmol) of 7-O-triethylsilylbacattine III **5** in 15 mL of anhydrous THF at −40 °C. The resulting solution was stirred at −40 °C for 40 min, and the reaction was quenched by adding 50 mL of conc. solution of ammonium chloride, and the product was extracted with ethyl acetate (5 × 50 mL). The organic solutions were combined, washed with brine, dried over sodium sulfate, and evaporated. Column chromatography on silica gel with *n*-hexane:ethyl acetate (3:1) as eluent gave desired **6b** in 33% yield (230 mg) as a pale-yellow solid. ^1^H NMR (DMSO-*d*_6_) δ 8.01 (d, *J* = 7.2 Hz, 2H), 7.70 (t, *J* = 7.4 Hz, 1H), 7.60–7.56 (m, 3H), 7.42 (d, *J* = 7.4 Hz, 2H), 7.38 (t, *J* = 7.7 Hz, 2H), 7.27 (t, *J* = 7.2 Hz, 1H), 6.25 (s, 1H), 5.93 (t, *J* = 8.9 Hz, 1H), 5.46 (d, *J* = 7.1 Hz, 1H), 5.39 (dd, *J* = 9.2, 7.6 Hz, 1H), 5.19 (br s, 1H), 5.11 (br s, 1H), 4.97 (d, *J* = 10.1 Hz, 1H), 4.80 (d, *J* = 7.3 Hz, 1H), 4.75 (s, 1H), 4.70 (br s, 1H), 4.67 (br s, 1H), 4.41 (dd, *J* = 6.8 Hz, 3.7 Hz, 1H), 4.39 (s, 5H), 4.08–4.05 (m, 2H), 3.67 (d, *J* = 7.1 Hz, 1H), 2.53–2.48 (m, 1H overlapped with DMSO-*d*_6_ residual peak), 2.44 (s, 3H), 2.12 (dd, *J* = 15.2, 9.4 Hz, 1H), 2.08 (s, 3H), 1.89 (dd, *J* = 15.2, 9.0 Hz, 1H), 1.70–1.65 (m, 1H), 1.65 (s, 3H), 1.55 (s, 3H), 1.05 (s, 3H), 1.02 (s, 3H), 0.88 (t, *J* = 8.0 Hz, 9H), 0.87 (t, *J* = 8.0 Hz, 9H), 0.59 (q, *J* = 7.9 Hz, 6H), 0.55–0.49 (m, 6H); ^13^C­{^1^H} NMR (DMSO-*d*_6_) δ 201.3, 171.4, 170.1, 168.8, 167.2, 165.1, 139.0, 138.4, 133.4, 133.3, 129.9, 129.5, 128.6, 128.1, 127.7, 127.6, 83.2, 80.5, 79.9, 76.7, 75.3, 74.6, 74.5, 74.4, 72.0, 71.9, 71.8, 71.2, 70.5, 70.2, 69.9, 57.6, 55.5, 45.9, 42.9, 36.7, 34.7, 26.2, 22.4, 21.1, 20.7, 20.4, 14.0, 13.7, 9.7, 6.4, 4.7, 4.1; Elemental analysis calcd for C_63_H_83_NO_14_RuSi_2_ C 61.24, H 6.77, N 1.13 found C 61.09, H 6.77, N 1.11.

#### *N*-(Adamantan-1-oyl)-2′,7-O-bis­(triethylsilyl)-*N*-debenzoylpaclitaxel **6c**

This compound was synthesized in 60% yield (450 mg of white powder) by the same procedure as described for **6b**, starting from **4c** instead of **4b**. ^1^H NMR (DMSO-*d*_6_) δ 8.01 (d, *J* = 7.2 Hz, 2H), 7.70 (t, *J* = 7.4 Hz, 1H), 7.60 (d, *J* = 7.8 Hz, 1H), 7.57 (d, *J* = 9.0 Hz, 1H), 7.42 (d, *J* = 7.4 H, 2H), 7.38 (t, *J* = 7.7 Hz, 2H), 7.28 (d, *J* = 7.2 Hz, 1H), 7.25–7.23 (m, 1H), 6.29 (s, 1H), 5.91 (t, *J* = 8.9 Hz, 1H), 5.48 (d, *J* = 7.2 Hz, 1H), 5.36 (dd, *J* = 9.0, 5.8 Hz, 1H), 4.97 (d, *J* = 9.8 Hz, 1H), 4.77 (d, *J* = 5.8 Hz, 1H), 4.73 (s, 3H), 4.41 (dd, *J* = 10.4, 6.8 Hz, 1H), 4.07 (br s, 2H), 3.67 (d, *J* = 7.2 Hz, 1H), 3.29 (s, 1H), 2.50–2.47 (m, 1H), 2.42 (s, 3H), 2.13–2.09 (m, 1H), 2.11 (s, 3H), 1.96 (br s, 3H), 1.92–1.88 (m, 1H), 1.79 (br s, 6H), 1.77 (s, 3H), 1.69–1.63 (m, 8H), 1.56 (s, 3H), 1.08 (s, 3H), 1.05 (s, 3H), 0.88 (t, *J* = 8.0 Hz, 9H), 0.85 (t, *J* = 8.0 Hz, 9H), 0.58–0.48 (m, 12H); ^13^C­{^1^H} NMR (DMSO) δ 201.2, 176.2, 171.7, 170.0, 168.8, 165.2, 139.1, 138.7, 133.4, 133.3, 129.9, 129.5, 128.6, 128.1, 127.4, 127.2, 83.1, 79.9, 76.8, 75.3, 74.7, 74.5, 74.4, 71.9, 70.6, 57.6, 55.1, 45.9, 42.9, 38.5, 38.2, 36.7, 36.0, 34.4, 27.5, 26.1, 22.4, 21.1, 20.4, 13.7, 9.7, 6.4, 4.7, 4.0. Elemental analysis calcd for C_63_H_89_NO_14_Si_2_ C 66.34, H 7.87, N 1.23 found C 68.65, H 8.40, N 0.59.

#### *N*-Debenzoyl-*N*-ruthenocenoylpaclitaxel **1b**

2.0 mL of hydrogen fluoride in pyridine (70% HF) was added at RT to a solution of 200 mg (0.162 mmol) of **6b** in 7.0 mL of anhydrous acetonitrile, and 14 mL of pyridine was placed in a Teflon flask, and the resulting solution was stirred at RT for 20 h. Next, the reaction was quenched by adding 200 mL of sat. solution of sodium bicarbonate and the product was extracted with ethyl acetate (5 × 50 mL). The organic solutions were combined, washed with water, brine, dried over sodium sulfate, and evaporated to dryness. Column chromatography on silica gel with dichloromethane–methanol gradient starting from 0 to 5% of methanol gave 130 mg (80% yield) of **1b** isolated as a yellow powder. ^1^H NMR (DMSO-*d*_6_) δ 7.99 (d, *J* = 7.2 Hz, 2H), 7.88 (d, *J* = 8.8 Hz, 1H), 7.70 (t, *J* = 7.4 Hz, 1H), 7.62 (t, *J* = 7.7 Hz, 2H), 7.39 (t, *J* = 7.6 Hz, 2H), 7.33 (d, *J* = 7.4 Hz, 2H), 7.25 (t, *J* = 7.3 Hz, 1H), 6.32 (s, 1H), 6.02 (d, *J* = 8.3 Hz, 1H), 5.96 (t, *J* = 8.8 Hz, 1H), 5.45 (d, *J* = 7.2 Hz, 1H), 5.34 (dd, *J* = 8.6, 6.8 Hz, 1H), 5.21 (br s, 1H), 5.19 (br s, 1H), 4.92 (d, *J* = 9.9 Hz, 1H), 4.88 (d, *J* = 7.0 Hz, 1H), 4.74 (s, 1H), 4.70 (br s, 2H), 4.57 (dd, *J* = 8.1, 6.7 Hz, 1H), 4.52 (s, 5H), 4.13 (dq, *J* = 10.9, 6.8 Hz, 1H), 4.05 (d, *J* = 8.2 Hz, 1H), 4.01 (d, *J* = 8.2 Hz, 1H), 3.65 (d, *J* = 7.1 Hz, 1H), 2.36–2.31 (m, 1H), 2.23 (s, 3H), 2.12 (s, 3H), 2.00 (dd, *J* = 15.3, 9.2 Hz, 1H), 1.90 (dd, *J* = 15.3, 9.0 Hz, 1H), 1.83 (s, 3H), 1.67–1.63 (m, 1H), 1.52 (s, 3H), 1.40 (s, 3H), 1.05 (s, 6H); ^13^C­{^1^H} NMR (DMSO-*d*_6_) δ 202.3, 172.6, 169.8, 168.7, 167.3, 165.1, 149.5, 139.5, 139.3, 133.3, 129.9, 129.5, 128.6, 128.1, 127.2, 123.8, 83.5, 80.3, 80.2, 76.8, 75.3, 74.7, 74.5, 73.3, 71.9, 71.4, 70.4, 70.3, 70.0, 69.7, 57.4, 55.2, 46.1, 42.9, 36.5, 34.8, 28.9, 26.3 (2xC), 22.3, 21.3, 20.6, 13.8, 9.7. Elemental analysis calcd for C_51_H_55_NO_14_Ru C 60.83, H 5.51, N 1.39 found C 60.66; H 5.64; N 1.38. HPLC-MS *R*_f_ = 8.47 min, *m*/*z* calcd for C_51_H_56_NO_14_Ru [M + H]^+^ 1008.3 found 1008.0 [M + H]^+^.

#### *N*-(Adamantan-1-oyl)-*N*-debenzoylopaclitaxel **1c**

This compound was synthesized in 94% yield (300 mg of white powder) by the same procedure as that described for **1b**, starting from 400 mg (0.350 mmol) of **6c** instead of **6b**. ^1^H NMR (DMSO-*d*_6_) δ 8.58 (d, *J* = 4.1 Hz, 1H), 7.99 (d, *J* = 7.3 Hz, 2H), 7.69 (t, *J* = 7.4 Hz, 1H), 7.63–7.54 (m, 3H), 7.40–7.35 (m, 3H), 7.29 (d, *J* = 7.5 Hz, 2H), 7.23 (t, *J* = 7.3 Hz, 1H), 6.32 (s, 1H), 5.93–5.90 (m, 2H), 5.45 (d, *J* = 7.2 Hz, 1H), 5.29 (dd, *J* = 8.4, 6.4 Hz, 1H), 4.91 (d, *J* = 10.1 Hz, 1H), 4.88 (d, *J* = 6.9 Hz, 1H), 4.71 (s, 1H), 4.55 (dd, *J* = 7.8, 6.4 Hz, 1H), 4.14–4.10 (m, 1H), 4.04 (d, *J* = 7.9 Hz, 1H), 4.01 (d, *J* = 8.2 Hz, 1H), 3.64 (d, *J* = 7.2 Hz, 1H), 2.35–2.30 (m, 1H), 2.22 (s, 3H), 2.12 (s, 3H), 2.02–1.99 (m, 1H), 1.96 (br s, 4H), 1.93–1.89 (m, 1H), 1.84 (s, 3H), 1.80 (br s, 6 H), 1.69–1.63 (m, 8H), 1.52 (s, 3H), 1.06 (s, 6H). ^13^C­{^1^H} NMR (DMSO-*d*_6_) δ 202.3, 176.2, 172.7, 169.7, 168.7, 165.2, 149.5, 139.6, 139.2, 136.1, 133.3 (2xC), 129.9, 129.5, 128.6, 128.1, 127.0, 126.9, 123.8, 83.5, 80.2, 76.8, 75.3, 74.8, 74.5, 73.3, 70.4, 69.9, 57.4, 54.8, 46.1, 42.9, 38.5, 36.5, 36.0, 34.8, 30.9, 27.6, 26.2, 22.2, 21.2, 20.6, 13.8, 9.7. Elemental analysis calcd for C_51_H_61_NO_14_ C 67.16, H 6.74, N 1.54 found C 67.11, H 6.88, N 1.82. HPLC-MS R_f_ = 8.95 min, *m*/*z* calcd for C_51_H_62_NO_14_ [M + H]^+^ 912.4 found 912.8 [M + H]^+^, 934.7 [M + Na]^+^, 975.5 [M+MeCN + Na]^+^.

### Cell Culture

A panel of colon cancer cell lines (LS180, HCT116, SW620, and LoVo) and healthy colon cells CCD841 CoN were purchased from ATCC via LGC Standards. LS180 cells originate from a primary colon adenocarcinoma and correspond to stage II disease, whereas HCT116 cells were also derived from a primary tumor but classified as stage III. SW620 cells were established from a lymph node and represent a stage III tumor. Finally, LoVo cells were isolated from a liver metastasis and represent stage IV colorectal cancer. CCD841 CoN is an adherent cell line isolated from the colon tissue of a healthy donor. The multidrug-resistant SW620 V cell line was derived from parental SW620 cells as described previously.^69^ All cell lines were grown in standard cell culture conditions (100% relative humidity, 5% CO_2_, 37 °C) in high-glucose DMEM supplemented with GlutaMAX, HEPES, and 10% heat-inactivated fetal bovine serum (FBS). All media and supplements were obtained from Life Technologies (Paisley, UK). Adequate care was taken not to cross-contaminate the cultures (the cultures were processed separately using filter pipet tips). Cultures were monitored every 3 months for *Mycoplasma sp.* infection using a MycoProbe Mycoplasma Detection Kit (R&D Systems).

### Patient Cohort

Tissue specimens from 9 patients with colorectal cancer were obtained from CRC surgery at the Norbert Barlicki Memorial Teaching Hospital No. 1 of the Medical University of Lodz, Lodz, Poland, between June 2023 and September 2023. For immunohistochemical staining, the tissues were fixed in 10% neutral buffered formalin and embedded in paraffin. The specific clinicopathological data of the patients are presented in Table S1. The study was approved by the Ethical Committee of the Medical University of Lodz (No RNN/135/23/KE), and all patients provided written informed consent.

### Immunohistochemistry (IHC)

Immunohistochemical reactions were performed on 4 μm paraffin sections obtained from TMA blocks mounted on Superfrost Plus slides (Menzel Gläser, Braunschweig, Germany). The sections were dewaxed and rehydrated, and the epitopes were exposed using a Pre-Treatment Link Rinse Station and Target Retrieval Solution (pH 6 for *K*_i_-67; pH 9 for p16 and SATB1; 97 °C, 20 min) (Dako, Glostrup, Denmark). The activity of endogenous peroxidase was blocked by 5 min of exposure to the Peroxidase-Blocking Reagent (Dako). All slides were counterstained with Mayer’s hematoxylin, and the cancer progression and stages were characterized.

### Colon Cancer Cell Line Isolation from Patient Tissue

The aseptic solid cancer fragments obtained from inside the tumor tissue were excised carefully to avoid blood vessels and fatty acid elements and, after rapid transport, transferred to DMEM supplemented with penicillin (500 U/mL) and streptomycin (300 μg/mL) to prevent bacterial contamination with heparin (5 μg/mL). Next, the tumor tissue was placed in sterile PBS and carefully rinsed. Then, the necrotic areas, fatty tissue, and blood clots were removed with sterile scissors and forceps. The obtained tissue was minced with scissors and vigorously pipetted with 10 mL of PBS. After heavier pieces sedimentation, the aggregates were harvested, transferred into a 15 mL sterile tube, and centrifuged (400*g* for 5 min). The supernatant was discarded, and the tissue fragments were rewashed in PBS, centrifuged, and finally resuspended in 5 mL of DMEM supplemented with collagenase H (1 mg/mL, Sigma-Aldrich). The cells were placed in 25 cm^2^ flasks and maintained in a humidified atmosphere of 5% CO_2_ at 37 °C. After 3 days, the cells were trypsinized and transferred to new 25 cm^2^ flasks (1:2 division) in DMEM supplemented with 5% FBS, penicillin (100 U/mL), and streptomycin (100 μg/mL). Then, the cells were grown under standard conditions, and the medium was replaced 2 to 3 times per week. After 2 weeks of cell culture stabilization, the medium was changed to DMEM supplemented with 10% FBS, penicillin (100 U/mL), and streptomycin (100 μg/mL). All media and supplements were obtained from Life Technologies (Paisley, UK).

### Spheroid Culture

Colon cancer 3D spheroids were formed using Greiner Bio-One’s Magnetic 3D Cell Culture Technology according to the manufacturer’s instructions. Briefly, cells were grown to 80% confluence and labeled with NanoShuttle-PL (Greiner, Bio-One) overnight in a 6-well plate. Then, 30,000 cells were seeded into 96-well cell-repellent Greiner Bio-One flat-bottom plates and placed on a magnetic drive for 30 min. Next, the spheroids were treated with compounds or vehicle (1 μM or 0.25% DMSO), followed by incubation for 72 h to form 3D structures. The medium was replaced with fresh medium every 72 h until the end of the experiment (8 days). The spheroids were visualized each day using a light inverted Nikon D-Eclipse Ti microscope (4× objective) with Nikon NIS-Elements and analyzed using ImageJ software v. 1.53a.

### Interference RNA

A mixed set of four siRNAs targeting human TUBB4 and a negative control siRNA (scramble) were employed (Dharmacon, Lafayette, CO). The concentration of siRNA used was previously described.^70^ The siRNAs were transfected into cells with XfectTM Transfection Reagent (Clontech, Mountain View, CA) according to the manufacturer’s protocol. After the cells were silenced for 48 h, the silencing effect was confirmed each time through Western blot analysis.

### Transient Plasmid Transfection

An expression vector containing *TUBB3* (pcDNA3.1-TUBB3) and an empty vector (pcDNA3.1) were introduced into SW620 cells with Xfect Transfection Reagent (Clontech, Mountain View, CA) according to the manufacturer’s instructions. Briefly, exponentially growing cells in a 6-well plate were transfected with the pcDNA3.1-TUBB3 or pcDNA3.1 expression vector and cultured in medium supplemented with G418 (75 μg/mL) for 72 h. Next, the cells were subjected to an appropriate analysis. Moreover, TUBB3 overexpression was confirmed each time through Western blot analysis.

### Viability Assay

The sensitivity of colon cancer cell lines to new paclitaxel analogues was determined using the Sulforhodamine B assay as described previously.^60^ The results were calculated as a percentage of controls, and the IC_50_ values were calculated with GraphPad Prism v 9.0 software (GraphPad, Inc.) using four-parameter nonlinear logistic regression.

### Cell Cycle Analysis

Exponentially growing cells (1,00,000 cells/well seeded in 6-well plates 24 h before time 0) were treated with the IC_90_ of **1** and the molar equivalent of the tested compound for a specified time. The cells were then harvested by trypsinization, washed twice with ice-cold PBS, and fixed in 70% ethanol. After the cells were stored for at least 8 h at 4 °C, they were stained with propidium iodide staining solution (75 μM propidium iodide and 50 Kunitz units/ml of RNase A in PBS) for 30 min at 37 °C. The samples were analyzed using an LSRII (Becton Dickinson) instrument, and cell cycle phase distributions were determined with FlowJo 7.6.1 software (FlowJo, LLC) employing a built-in cell cycle analysis module (Watson pragmatic algorithm).

### Reactive Oxygen Species Production

The dihydrorhodamine 123 (DHR123) oxidation assay was performed as described previously.^38^ Exponentially growing SW620 cells (1,00,000 cells per well seeded in 6-well plates 20–24 h before time 0) were exposed to 1 μM of the tested compound for 4 h in the presence of 1 μM DHR123. The cells were then harvested by trypsinization and suspended in a complete growth medium. The samples were then analyzed using an LSRII (Becton Dickinson) instrument, and the median fluorescence (excitation 488 nm, emission 530/30 nm, FITC channel) was measured. The median fluorescence of cells incubated with DHR123 and DMSO alone was considered to be 100% in a given experiment.

### UIC2 Binding Assay

A total of 1,00,000 parental SW620 cells expressing low levels of ABC proteins or their MDR variant SW620 V cells overexpressing *ABCB1* were suspended in 100 μL of complete culture medium supplemented with solvent (DMSO), a positive control (vincristine), or an investigated compound at a final concentration of 10 μM. FITC-conjugated UIC2 anti-ABCB1 antibody was then added to a final concentration of 1 μg/mL, and the samples were incubated for 60 min at 37 °C. Then, the cells were washed three times (100*g* for 10 min) with fresh medium and analyzed using an LSRII (Becton Dickinson) instrument. The median fluorescence (excitation 488 nm, emission 530 nm, FITC channel) was collected and compared to a respective solvent control.

### Microtubule Isolation and Polymerization

Tubulin polymerization assay was performed using crude tubulin extracts isolated from drug-treated cancer cells to assess compound-induced changes in tubulin assembly in a cell-dependent context. Microtubule proteins were isolated from particular colon cancer cell lines as described previously.^71^ Next, the microtubules were suspended in G-PEM buffer (80 mM PIPES, pH 6.9, 1.0 mM GTP, 2 mM MgCl_2_, 0.5 mM ethylenediaminetetraacetic acid, and 5% glycerol) and incubated for 80 min in a 96-multiwell plate. The absorbance correlated with polymerization was measured at 340 nm every 4 min using a Synergy H4 Hybrid Microplate Reader (Agilent BioTek).

### Western Blot Analysis

Cell lysates and Western blotting were performed as described previously.^61^ The protein concentration was quantified with a BCA Protein Assay Kit according to the manufacturer’s protocol, and the protein lysates were stored at −70 °C until use. Densitometry data were analyzed with ImageJ software version 1.53 (Bethesda, MD), and protein levels were normalized to GAPDH. The antibodies used in the present study were anti-ILK, anti-integrin β, and anti-GAPDH (Santa Cruz Biotechnology, Dallas, TX); anti-CD98, anti-TUBB3, and anti-TUBB4 (Sigma-Aldrich, Taufkirchen, Germany); and HRP-conjugated secondary antibodies (Dako, Ely, UK).

### Transwell Invasion Assay

The invasion assay was performed as described previously with modifications.^2^ To assess the invasive potential of cells, inserts with 8.0 μm pore-size polycarbonate membranes were employed (ThinCert, Greiner, Kremsmünster, Austria). Before cell seeding, the upper surface of each membrane was coated with a thin layer of growth factor-reduced Matrigel (Corning, NY), prepared according to the manufacturer’s instructions, and allowed to polymerize at 37 °C for 30 min. The lower chambers were filled with complete DMEM containing 20% fetal bovine serum (FBS) to provide a chemoattractant gradient. Next, cells were suspended in serum-free DMEM and seeded in the upper chambers. The plates were incubated for 8 h at 37 °C in a humidified incubator with 5% CO_2_, allowing the cells to invade through the Matrigel layer and migrate toward the serum-rich medium below. Then, cells were fixed with 4% paraformaldehyde in PBS, stained with 1% crystal violet, and visualized with an EVOS FLoid Cell Imaging Station (Thermo Fisher Scientific, Bothell, WA). The number of transmigrated cells in five representative fields was counted by ImageJ software, version 1.53 (Bethesda, MD).

### MMP-7 ELISA

MMP-7 secretion was analyzed with a Human Total MMP-7 Quantikine ELISA Kit (R&D Systems, Minneapolis, MN) according to the manufacturer’s protocol. The optical density was measured at 450 nm using an Infinite F50 microplate reader (Tecan, Groding, Austria). The MMP-7 level is presented as ng of MMP-7 secreted by 1 million cells.

### Fluorescence Microscopy

A total of 5000 cells/ml were grown on sterile glass microscope slides at 37 °C in a humidified atmosphere of 5% CO_2_. Following treatment, the cells were washed twice with PBS and fixed (4% formaldehyde in PBS; 20 min; room temperature). Then, the cells were washed three times with PHEM buffer (60 mM PIPES, pH 6.9; 25 mM HEPES; 10 mM EGTA; 4 mM MgCl_2_ supplemented with protease inhibitors (cOmplete)-EDTA-free protease inhibitor cocktail), permeabilized in 0.1% Triton X-100 (v/v) in PHEM (5 min; on ice), and blocked in 2% (v/v) BSA in PHEM buffer for 1 h. Next, the localization protein was detected at 37 °C for 60 min with the following antibodies: mouse TUBA antibodies conjugated to Alexa 488 and rabbit anti-integrin-β1 antibodies conjugated to Alexa 546 or rabbit antivinculin antibodies conjugated to Alexa 546. Then, the nuclei were stained with DAPI by incubation for 30 min. Images were captured with an EVOS FLoid Cell Imaging Station (Thermo Fisher Scientific, Bothell, WA).

### Statistical Analysis

All the data were analyzed using GraphPad Prism version 9.1 software (GraphPad, Inc., San Diego, CA). The data are shown as the mean ± standard deviation (SD) of at least three independent experiments. The statistical significance of the differences was assessed by one-way or two-way ANOVA and Tukey’s post hoc test, assuming 0.05 as the significance level.

## Supplementary Material





## Notes

Molecular formula strings and associated biological data (CSV)
Additional tables, figures, and copies of the NMR and ESI spectra (PDF)

## Abbreviations Used

1-Ad: adamant-1-yl
ABCB1: ATP binding cassette subfamily B member 1
BCA Protein Assay: Bicinchoninic acid Protein Assay
Col.: colchicine
DAPI: 4′,6-diamidino-2-phenylindole
DHR123: dihydrorhodamine 123
DMEM: Dulbecco’s modified Eagle’s medium
EMT: epithelial-to-mesenchymal transition
FA: focal adhesion
FAs: focal adhesion sites
Fc: ferrocenyl
FBS: fetal bovine serum
FITC: fluorescein isothiocyanate
G418: Geneticin
HEPES: 4-(2-hydroxyethyl)-1-piperazineethanesulfonic acid
ILK: integrin-linked kinase
LiHMDS: Lithium bis­(trimethylsilyl)­amide
Rc: ruthenocenyl
SD: standard deviation
TES: triethylsilyl
TMA blocks: tissue microarray block
TRITC: tetramethylrhodamine isothiocyanate
TUBA: α-tubulin
TUBB: β-tubulin
TUBB1: βI-tubulin
TUBB2: βII-tubulin
TUBB3: βIII-tubulin
TUBB4A: βIVa-tubulin
TUBB6: βVI-tubulin
UIC2 antibodies: P-glycoprotein monoclonal antibody
