# Supplementary material for: Ruthenocenyl and 1‑Adamantyl Paclitaxel Analogs Disrupt the Balance between βIII- and βIVa-Tubulin and Inhibit the Growth and Invasiveness of Colon Cancer
Source: J Med Chem. 2025 Sep 4;68(18):19062–75. doi: 10.1021/acs.jmedchem.5c01147 (PMC12481480; doi:10.1021/acs.jmedchem.5c01147)
Supplement: Supplementary file 2 [file jm5c01147_si_002.pdf]

Supporting Information

to

Ruthenocenyl and 1-Adamantyl Paclitaxel Analogs Disrupt the Balance Between βIII- and βIVa-Tubulin and Inhibit the Growth and Invasiveness of Colon Cancer

Wojciech M. Ciszewski,[1] Karolina Kowalczyk,[2] Andrzej Błauż,[3] Wojciech Ciesielski,[4] Piotr Hogendorf,[4] Beata Smolarz,[5] Waldemar Wagner,[6] Anna Wieczorek-Błauż,[7] Błażej Michał Rychlik,[3] Hanna Romanowicz,[5] Adam Durczyński,[4] Katarzyna Sobierajska, [1]* and Damian Plażuk[2]*

[1] Department of Molecular Cell Mechanisms, Medical University of Lodz, Mazowiecka 6/8, 92-215 Lodz, Poland

[2] Laboratory of Molecular Spectroscopy, Department of Organic Chemistry, Faculty of Chemistry, University of Lodz, Tamka 12, 91-403 Lodz, Poland

[3] Centre for Digital Biology and Biomedical Science - Biobank Lodz, Faculty of Biology and Environmental Protection, University of Lodz, ul. Pomorska 141/143, 90-236 Łódź, Poland

[4] Department of General and Transplant Surgery, Medical University of Lodz, Kopcinskiego 22, 90-419 Lodz, Poland.

[5] Laboratory of Cancer Genetics, Department of Pathology, Polish Mother's Memorial Hospital Research Institute, Rzgowska 281/289, 93-338 Lodz, Poland

[6] Laboratory of Cellular Immunology, Institute of Medical Biology PAS, Lodowa 106, 93-232 Lodz, Poland.

[7] Department of Organic Chemistry, Faculty of Chemistry, University of Lodz, Tamka 12, 91-403 Lodz, Poland

Corresponding Author Information

E-mail: damian.plazuk@chemia.uni.lodz.pl

E-mail: katarzyna.sobierajska@umed.lodz.pl

Table of Contents

Additional biological experimental data S3

Copies of the NMR spectra and MS spectra S10

Copies of HPLC chromatograms S17

Additional Biological Experimental Data

Table S1: Clinicopathological characteristics of colon cancer patients

Characteristic	Variable	Value
**Sex**	male	n = 6
	female	n = 3
**Age**		68.1 ± 6.6
**Tumor localization**	rectum	n = 4
	sigmoid	n = 2
	descending colon	n = 2
	ascending colon	n = 1
**AJCC stage**	I	n = 1
	II	n = 4
	III	n = 2
	IV	n = 2
**Histopatho. score**	1	n = 1
	2	n = 4
	>3	n = 4
**status**	low grade	n = 5
	high grade	n = 4

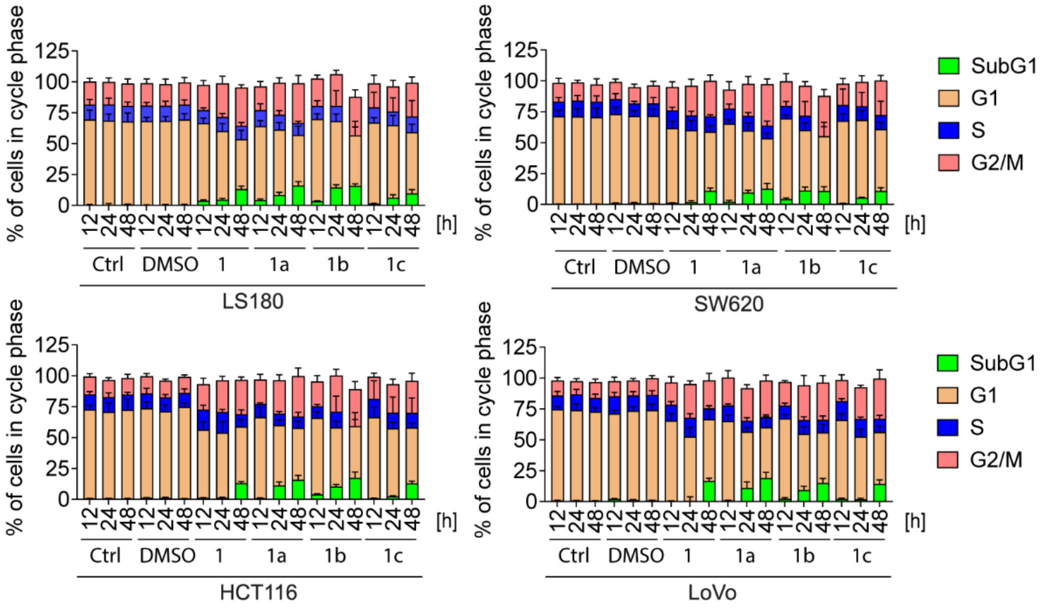

Figure S1. The cell cycle phase distribution in cells treated with **1** or **1a-c.** Graphs presenting the mean of % of cells in cycle phase ± SD (n = 3).

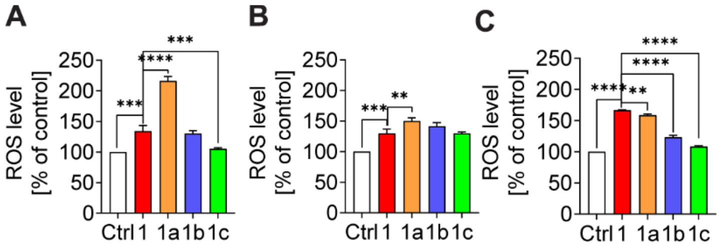

Figure S2. Reactive oxygen species (ROS) production in (A) LS180, (B) SW620 and (C) LoVo cells exposed to the investigated compounds. Graphs presenting the mean of fluorescence normalized to control and converted to percent values ± SD (n = 3), **p <0.01, *** p <0.005, ****p <0.001.

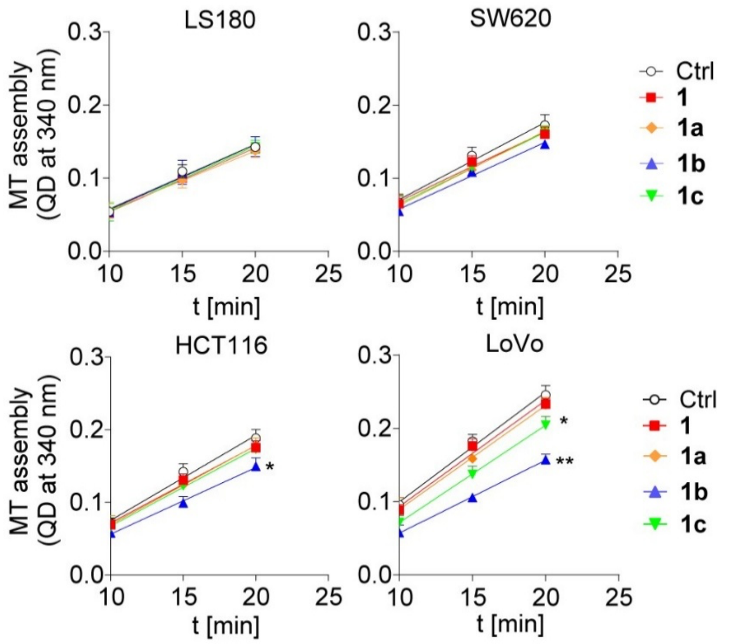

Figure S3. Initial slopes of tubulin (10 mg/mL) polymerisation curves from LS180, SW620, HCT116, and LoVo cells previously treated with compounds **1**, colchicine (Col.), and **1a–c** at 3 μM concentration. Slopes were estimated from the 10–20 min interval of each curve, corresponding to the exponential (elongation) phase, and indicate the speed of tubulin polymerisation. The graphs display mean ± S.D. (n = 3), $^*p < 0.05$, $^{**}p < 0.01$.

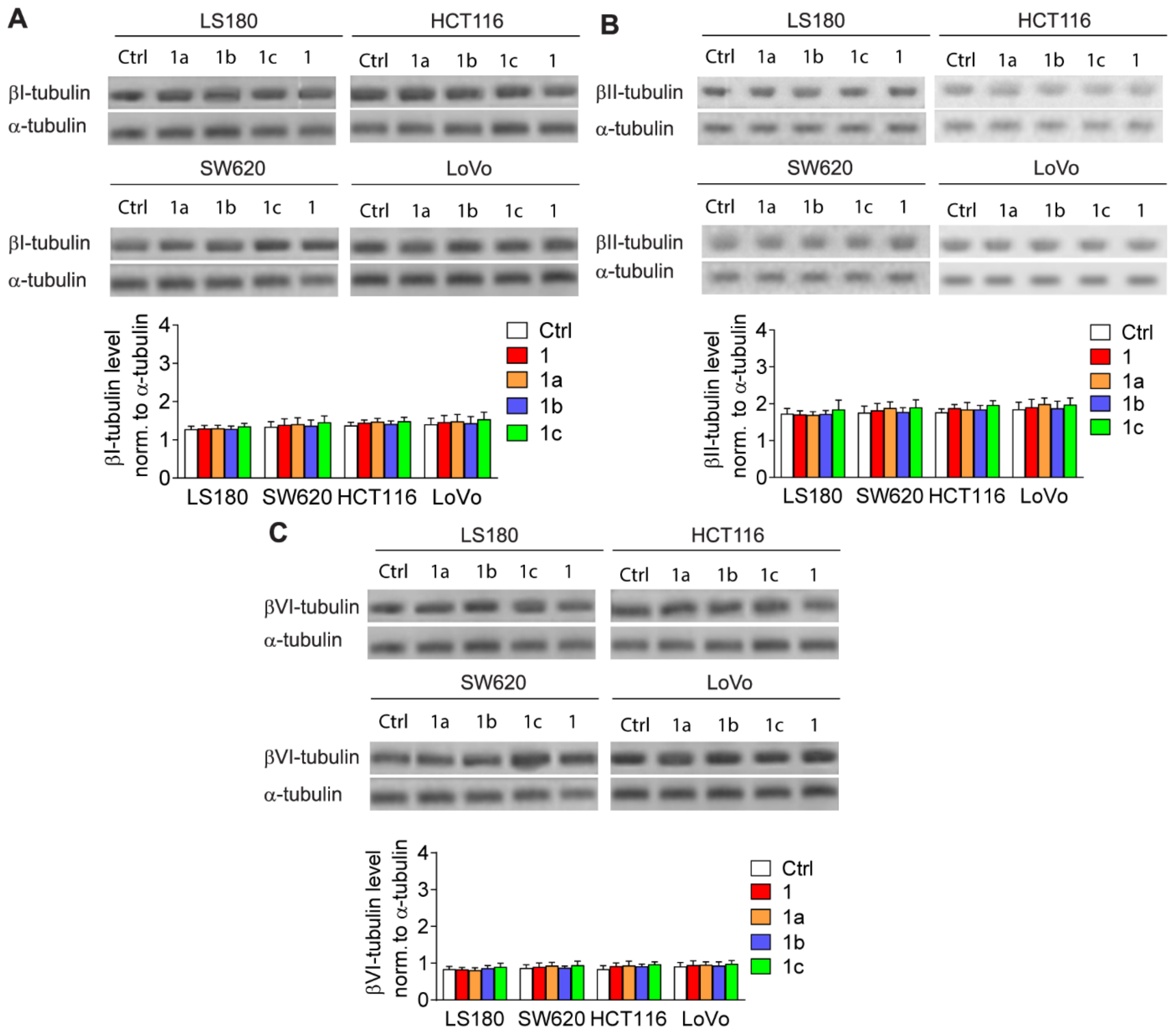

Figure S4. Modulation of (A) βI-tubulin, (B) βII-tubulin and (C) βVI-tubulin protein level in LS180, SW620, HCT 116 and LoVo cells treated with **1** or **1a-c.** Representative Western blot results are shown, graphs presenting the mean optical density of βI-tubulin, βII-tubulin or βVI-tubulin normalized to corresponding α-tubulin bands ± SD (n = 3).

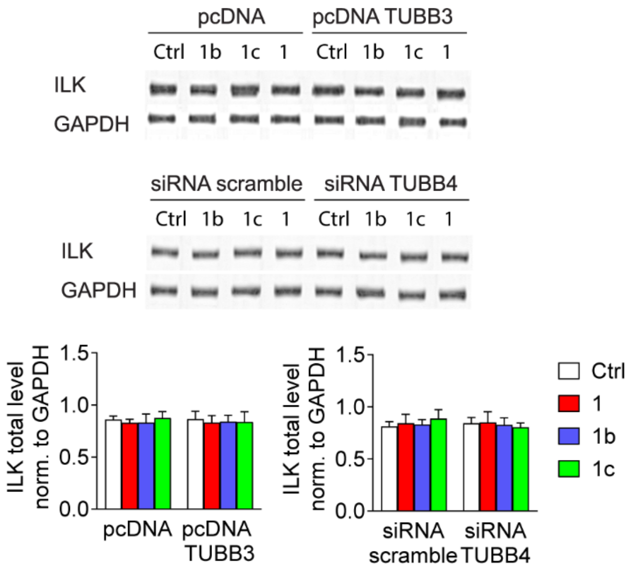

Figure S5. Modulation of total ILK in βIII-tubulin overexpressed or βIVa-tubulin silenced LoVo cells treated with **1** or its analogues **1b-c.** Representative Western blot results are shown, graphs presenting the mean optical density of cytosolic ILK normalized to GAPDH ± SD (n = 3).

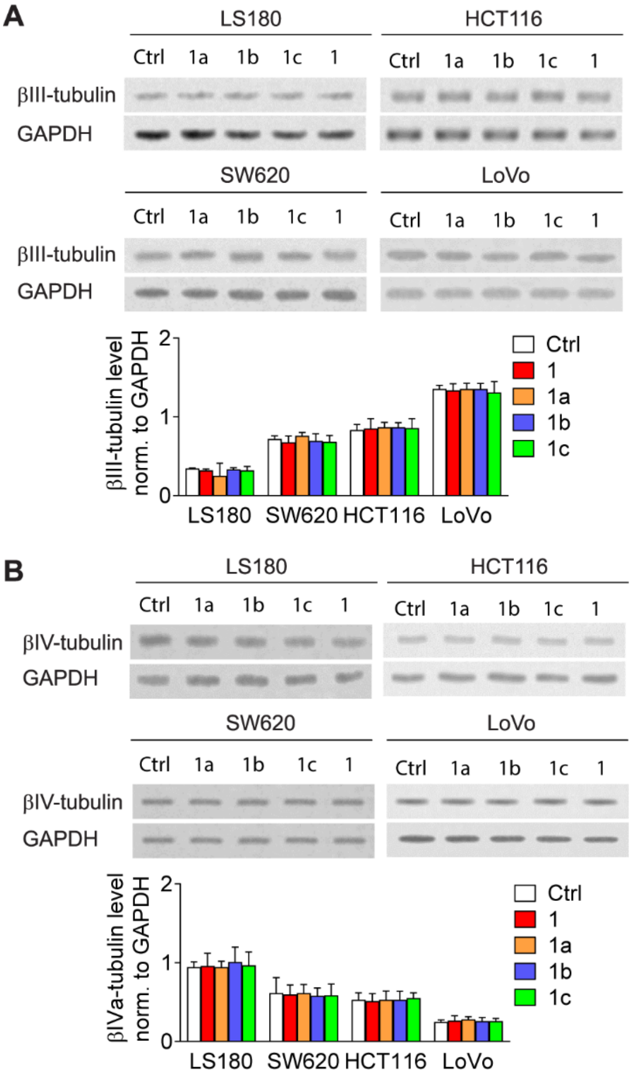

Figure S6. Alteration of total (A) βIII-tubulin and (B) βIVa-tubulin level in **1** or its analogues **1a-c** – short term-treated cells. Representative Western blot results are shown, graphs presenting the mean optical density of βIII-tubulin or βIVa-tubulin normalized to GAPDH ± SD (n = 3).

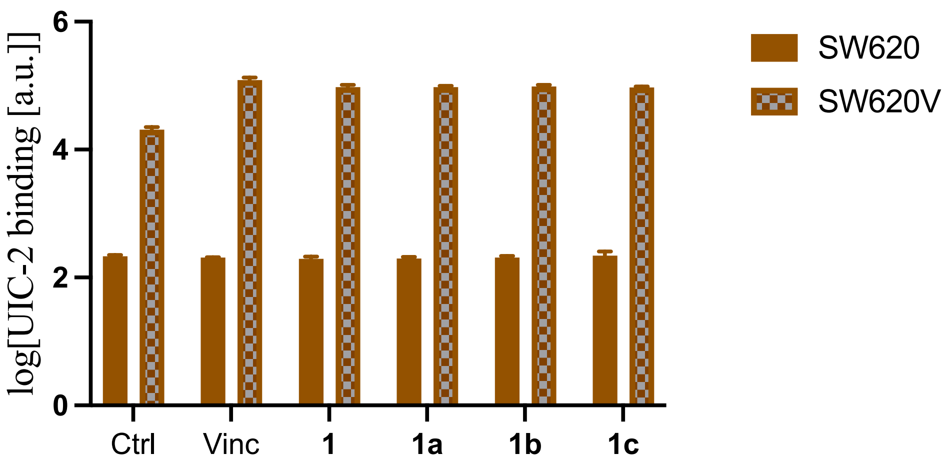

Figure S7. UIC2 monoclonal antibody binding to SW620 (solid bars) and SW620V (dotted bars) cells exposed to 10 µM of the investigated compounds indicating allocrite recognition by ABCB1. Vincristine (Vinc) was used as a model allocrite (a positive control). The graph presenting the log values of the mean of UIC2 binding ± SD (n = 3).

Copies of the NMR spectra and MS spectra

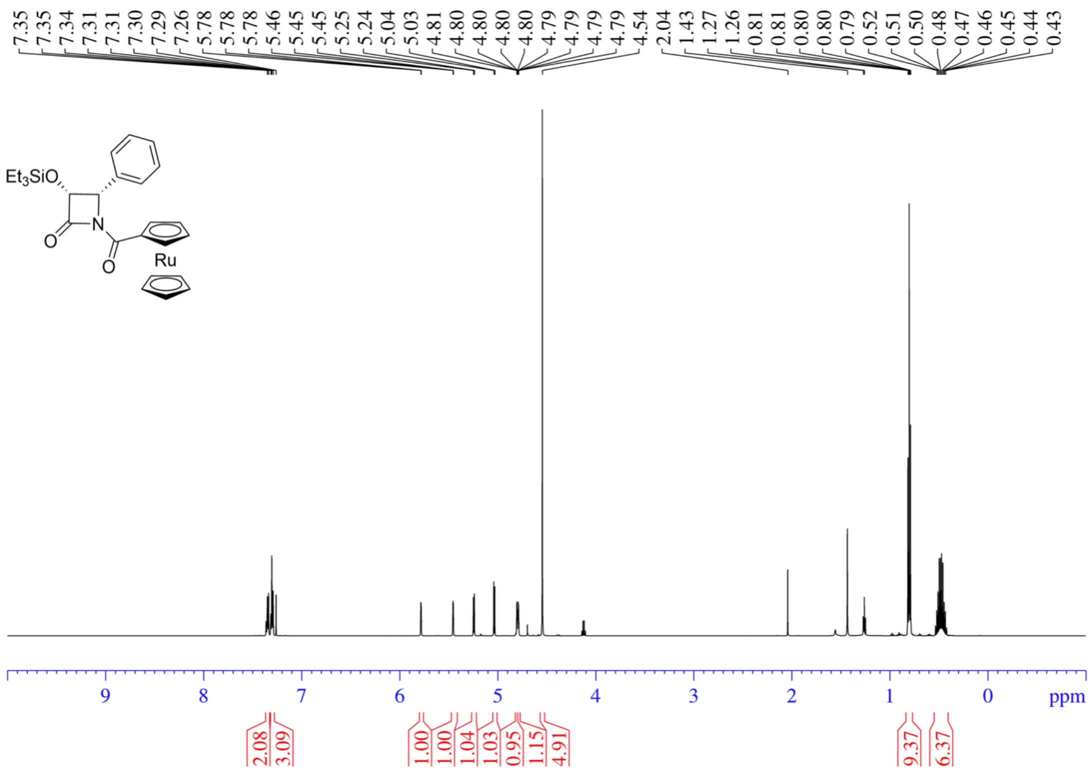

Figure S8. ^{1}H NMR spectrum of **4b** in $CDCl_3$

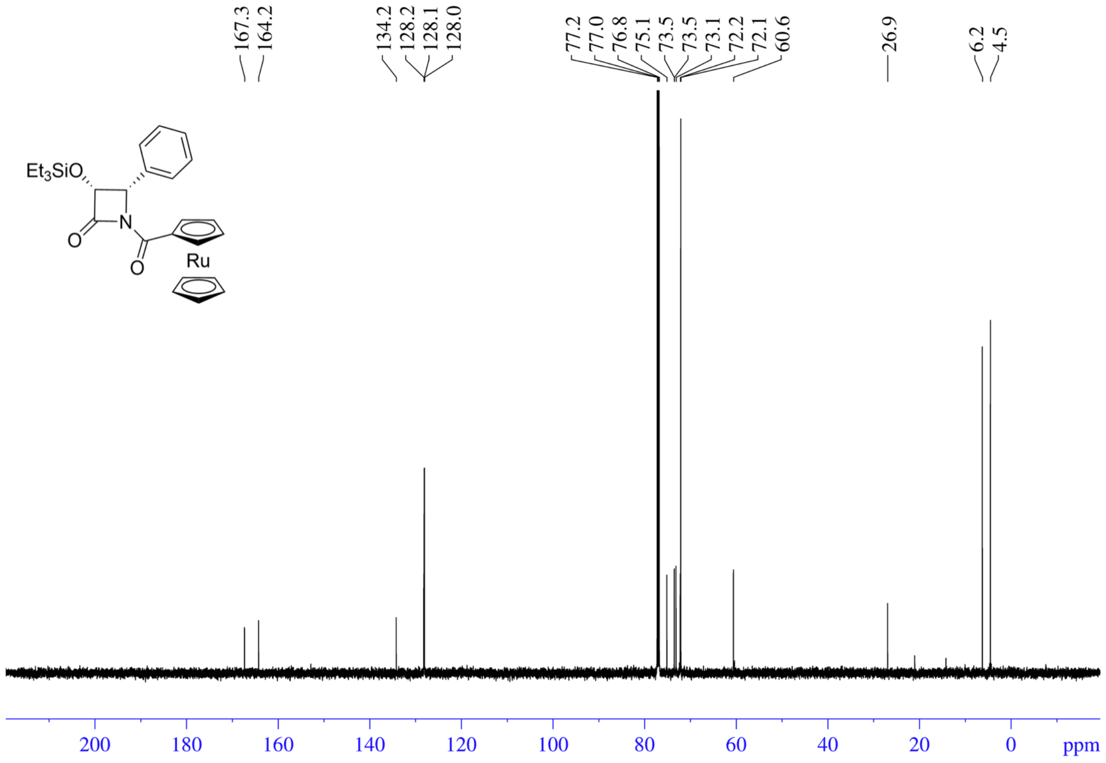

Figure S9. $^{13}C\{^{1}H\}$ NMR spectrum of **4b** in $CDCl_3$

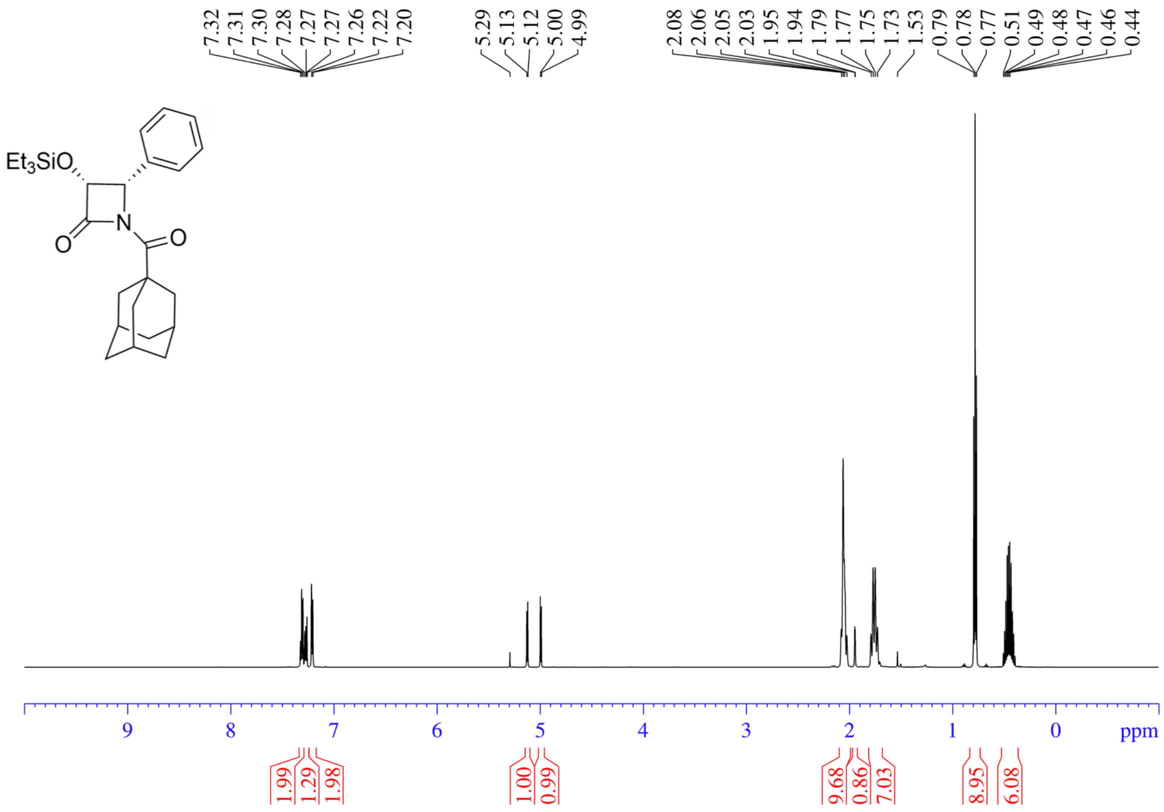

Figure S10. ^{1}H NMR spectrum of **4c** in $CDCl_3$

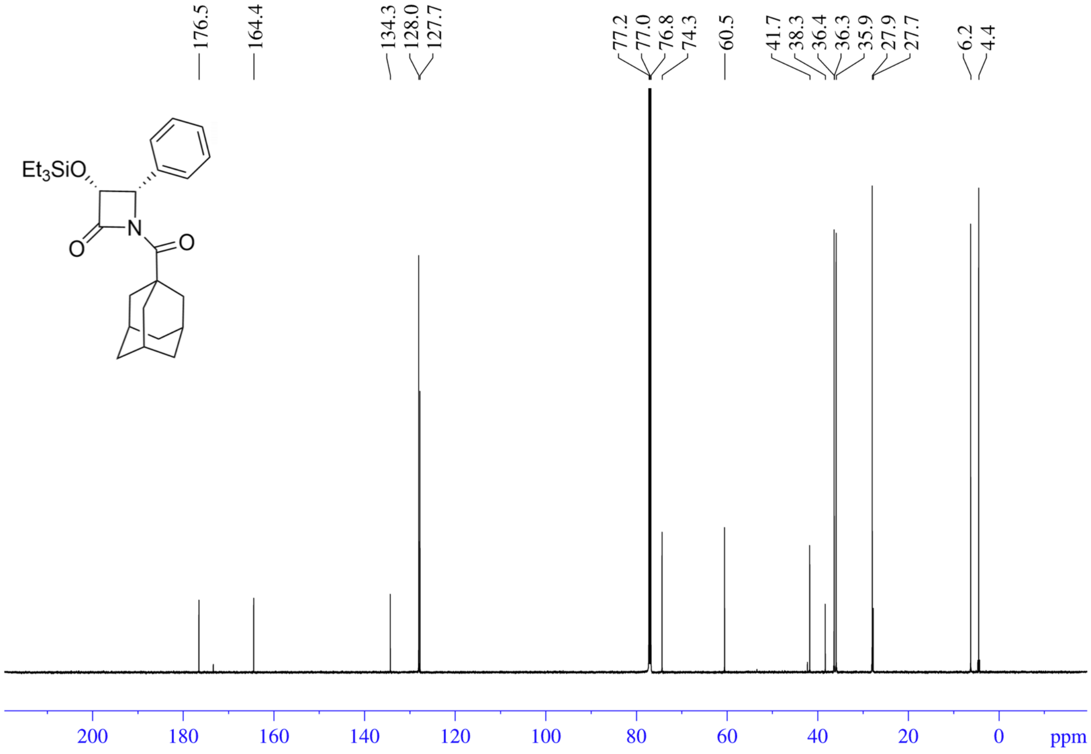

Figure S11. $^{13}C\{^{1}H\}$ NMR spectrum of **4c** in $CDCl_3$

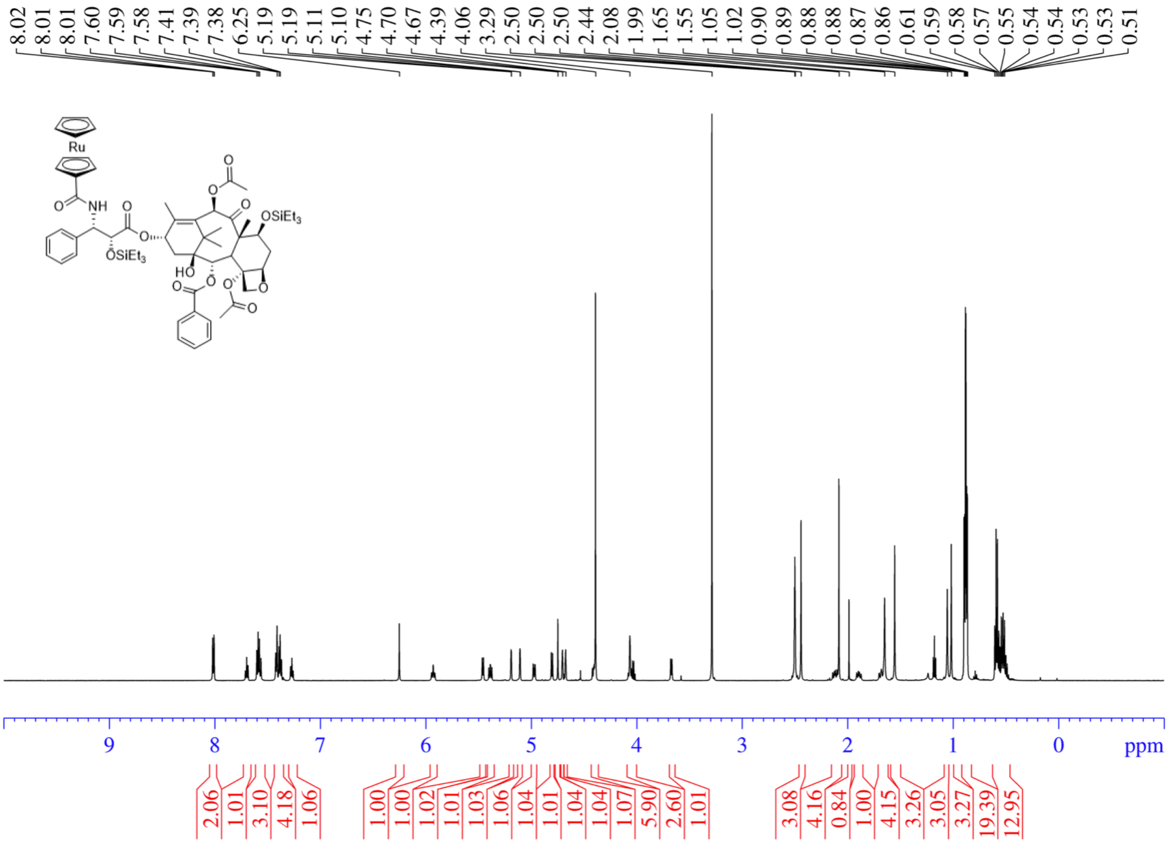

Figure S12. ^{1}H NMR spectrum of **6b** in DMSO-d_6

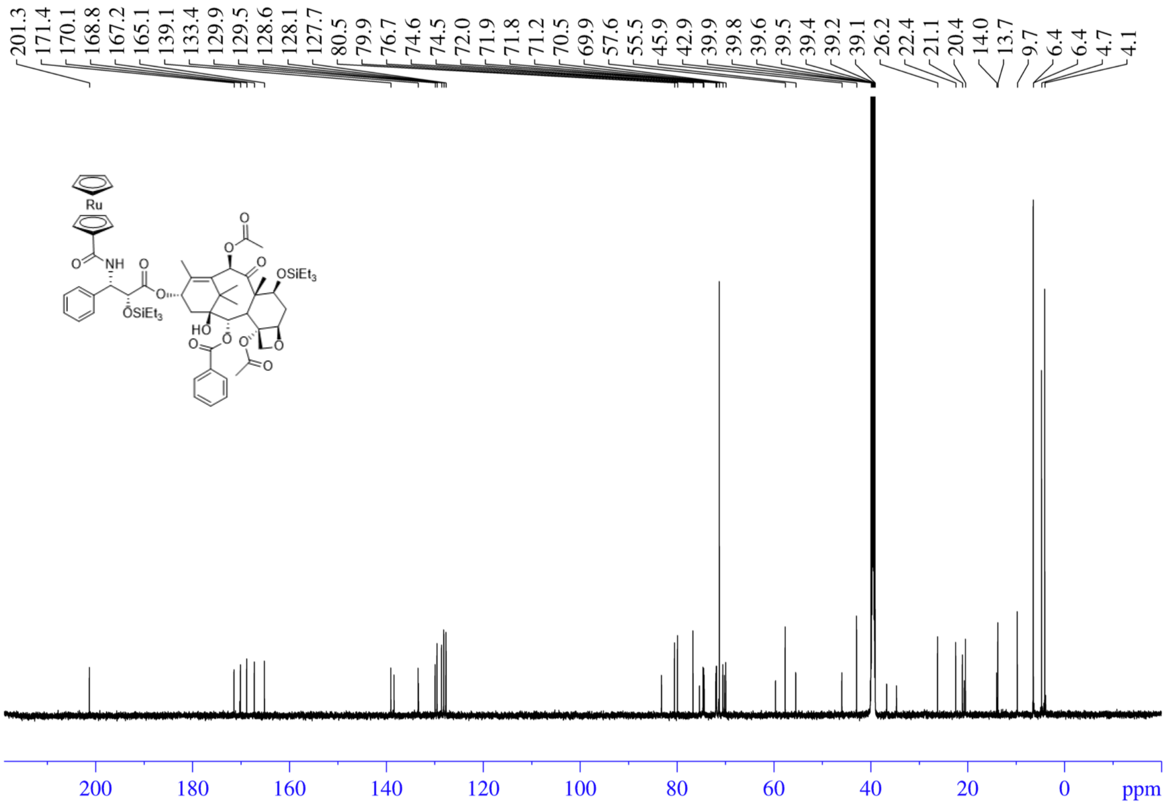

Figure S13. $^{13}C\{^{1}H\}$ NMR spectrum of **6b** in DMSO-d_6

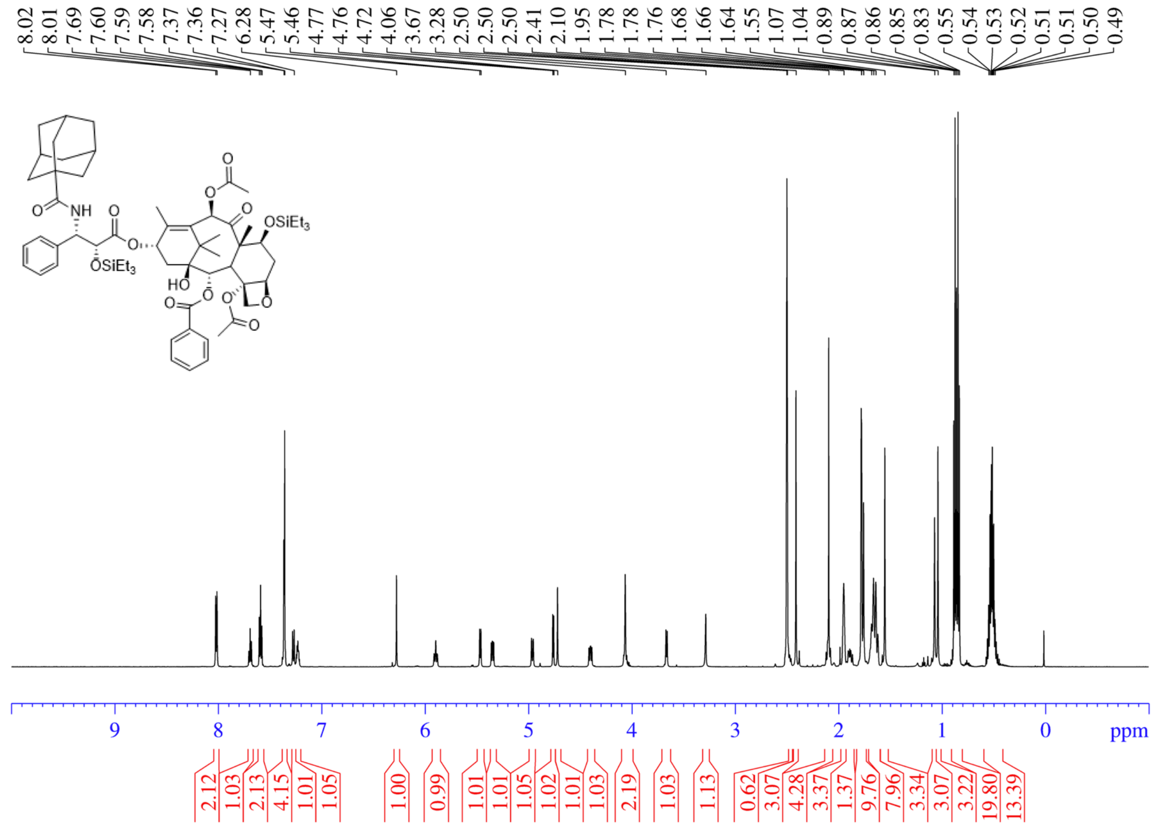

Figure S14. $^{13}C\{^1H\}$ NMR spectrum of **6c** in DMSO-d$_6$

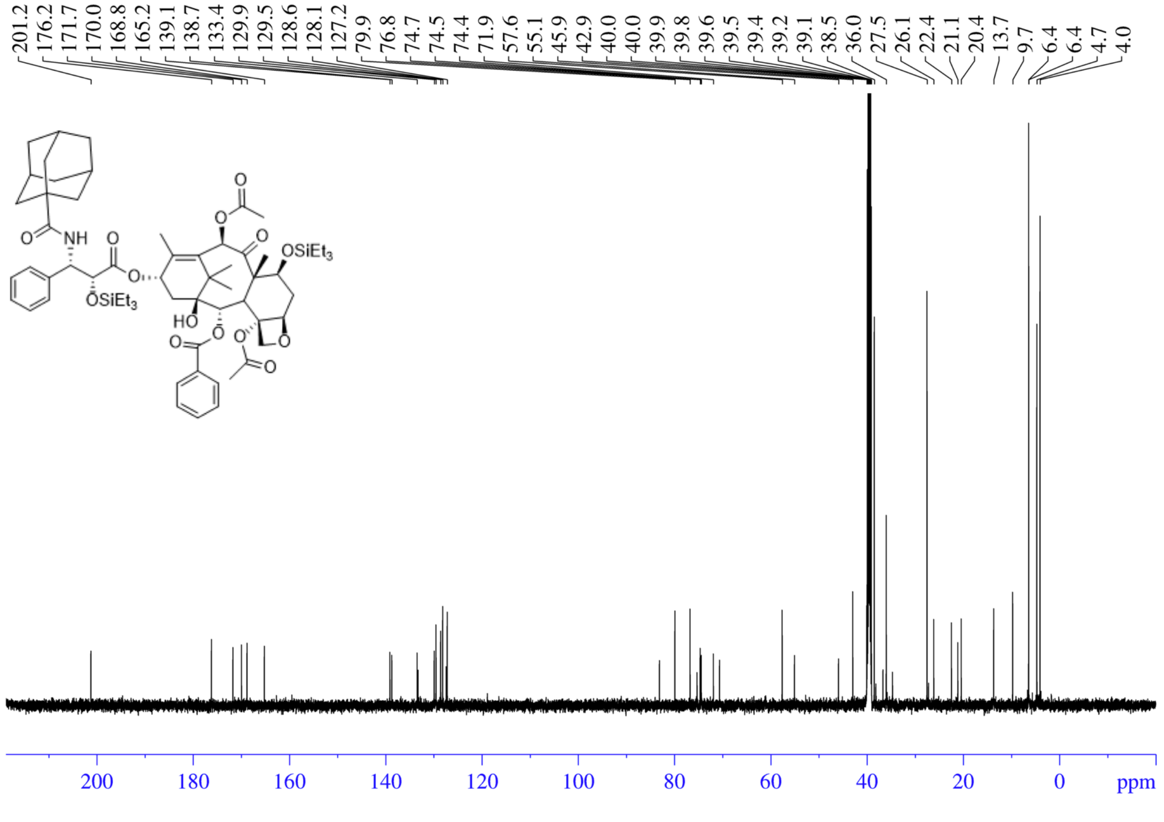

Figure S15. $^{13}C\{^1H\}$ NMR spectrum of **6c** in DMSO-d$_6$

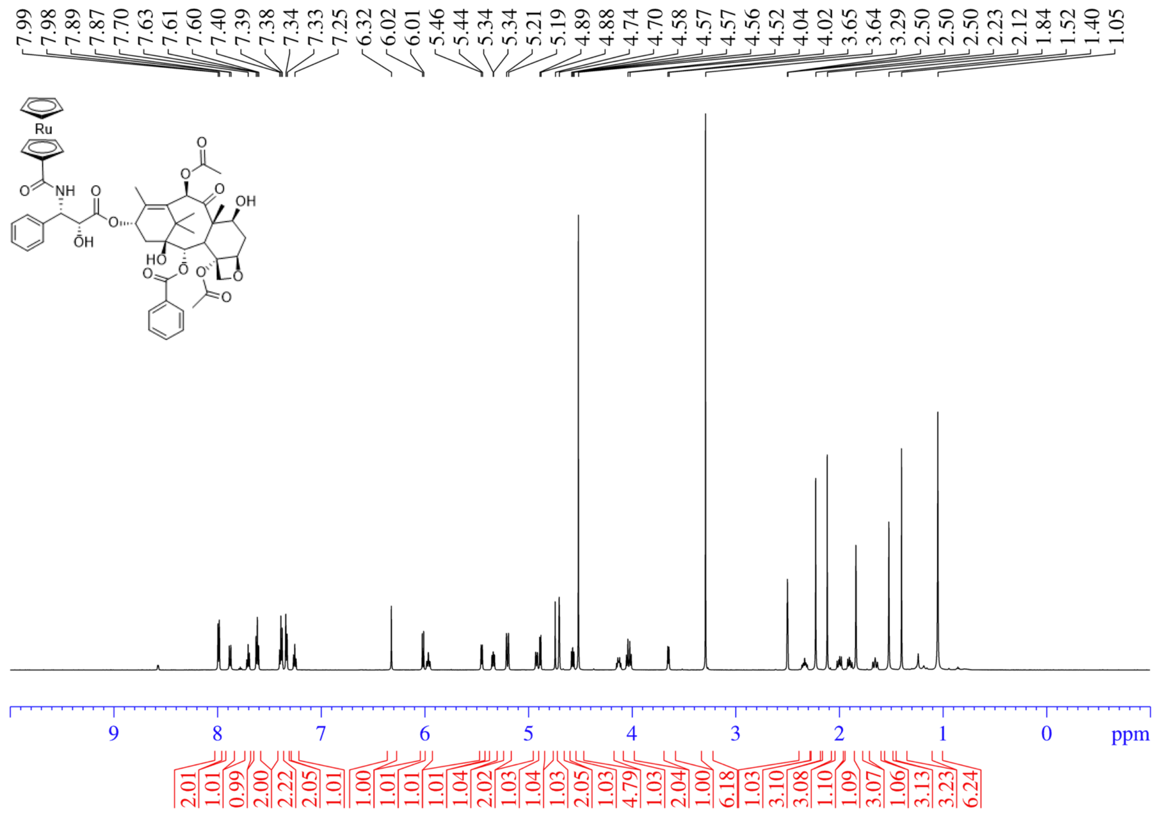

Figure S16. ^{1}H NMR spectrum of **1b** in DMSO-d$_6$

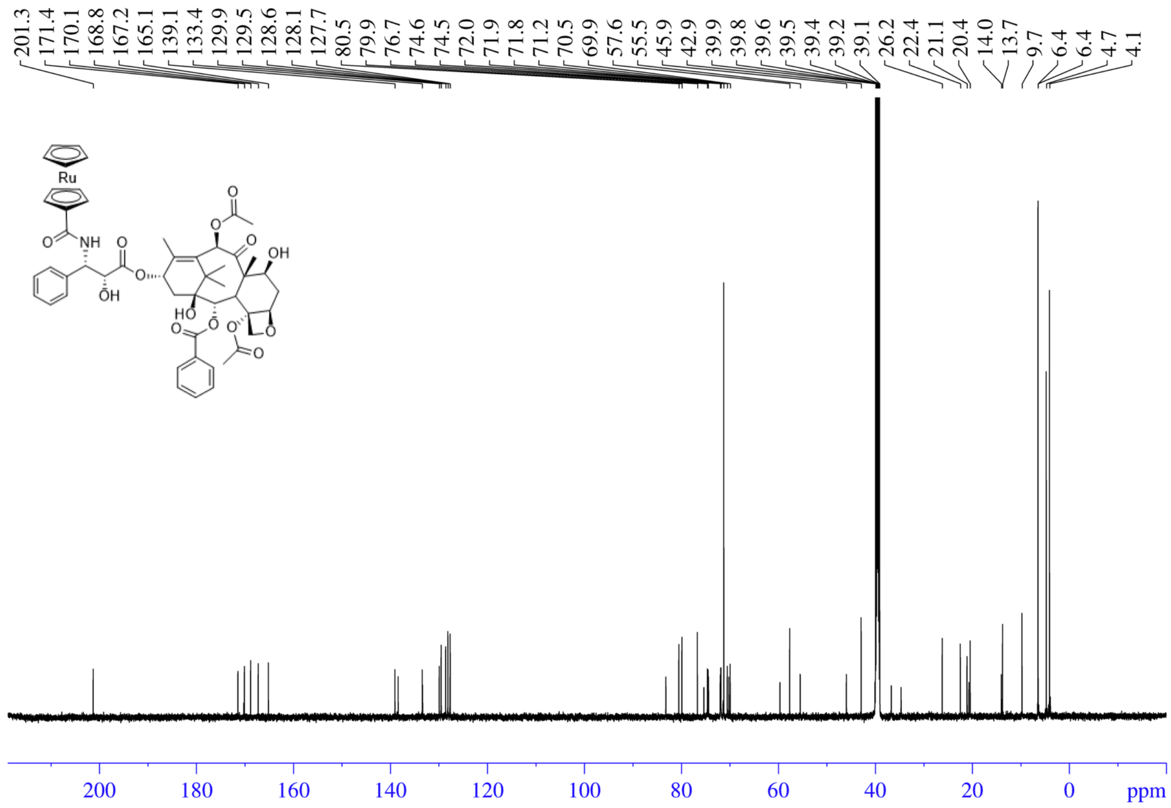

Figure S17. $^{13}C\{^{1}H\}$ NMR spectrum of **1b** in DMSO-d$_6$

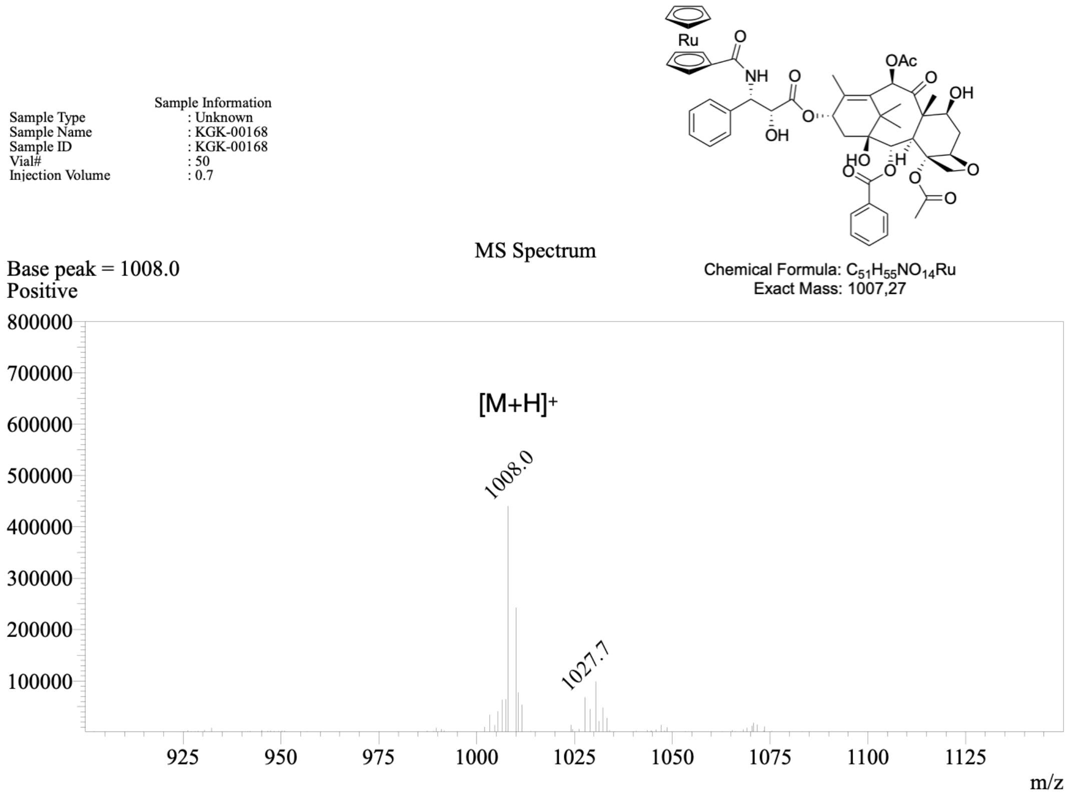

Figure S18. ESI-MS spectrum of **1b**

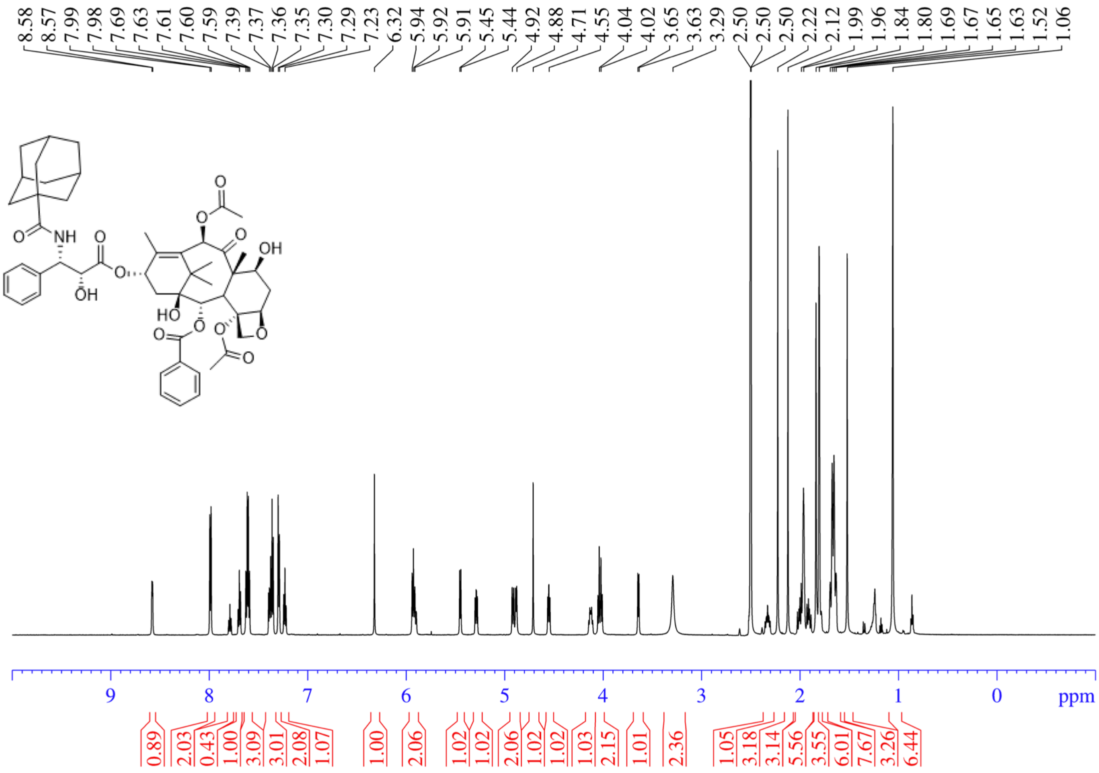

Figure S19. ^{1}H NMR spectrum of **1c** in DMSO-d$_6$

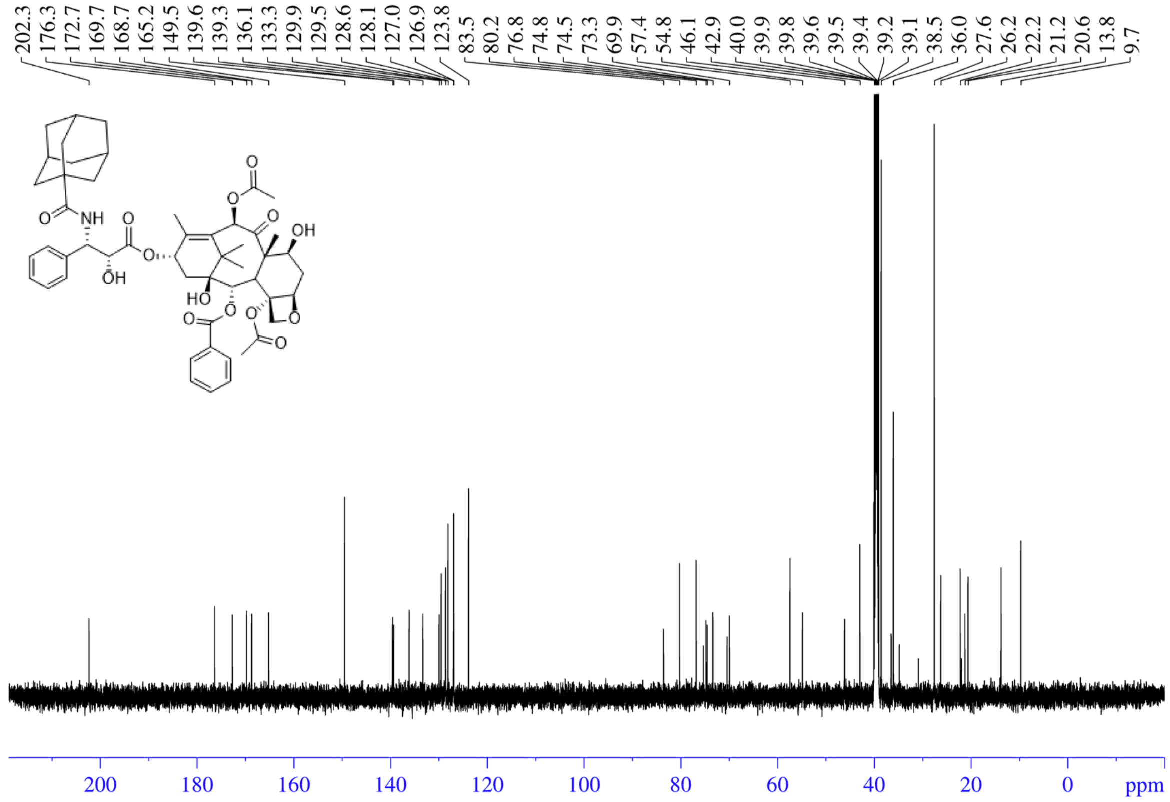

Figure S20. $^{13}C\{^{1}H\}$ NMR spectrum of **1c** in DMSO-d$_6$

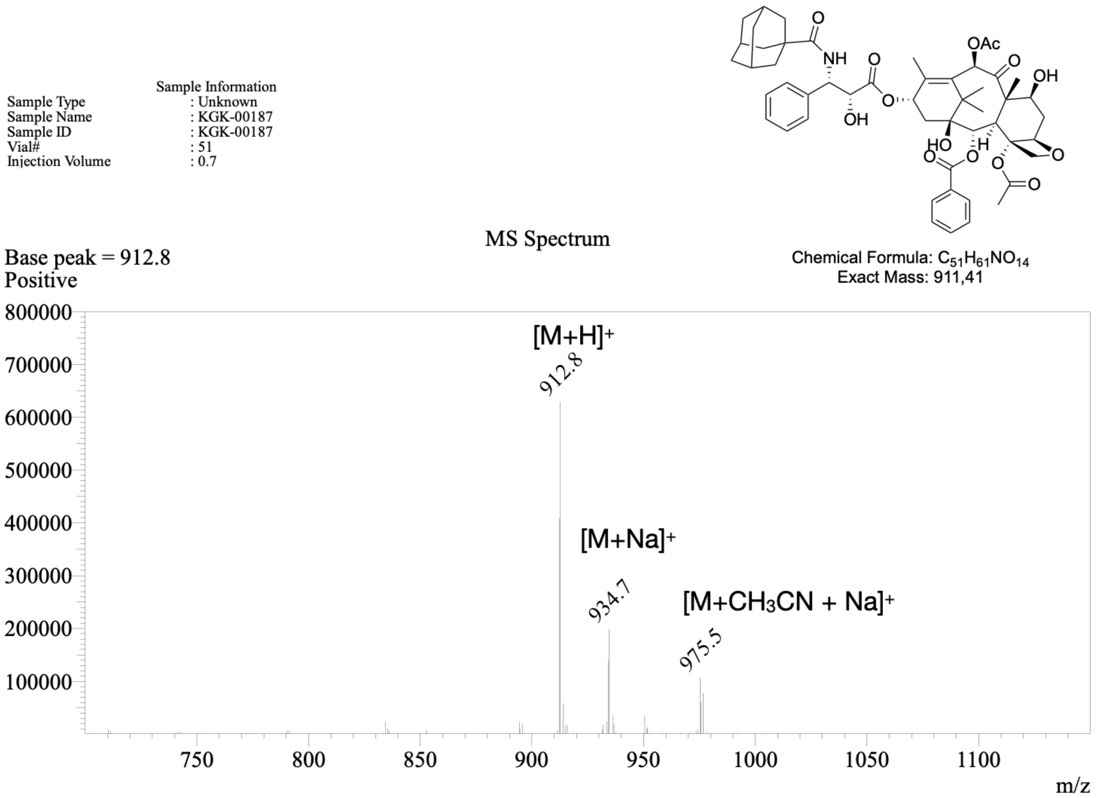

Figure S21. ESI-MS spectrum of **1c**

Copies of HPLC chromatograms

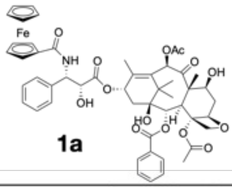

1a

Sample Information	
Sample Type	: Unknown
Sample Name	: DPX-KK-147
Sample ID	: DPX-KK-147
Vial#	: 1
Injection Volume	: 0.5

Method

<<LC Time Program>>

Time	Module	Command	Value
5.00	Pumps	B.Conc	90
6.00	Pumps	B.Conc	90
8.00	Pumps	B.Conc	30
15.00	Controller	Stop	

<<Column Infomation>>
Column Name : Kinetex XB-C18
Column Length : 50 mm
Inner Diameter : 2.1 mm
ParticleSize : 1.7 um

<<Mobile Phase Name>>
Mobile Phase A : water+0.01%HCOOH
Mobile Phase B : Methanol+0.01%HCOOH

<<Data Acquisition>>
LC Stop Time : 15.00 min

<<Pump>>
Total Flow : 0.3000 mL/min
B Conc. : 30.0 %

<<Autosampler>>

<<PDA>>
PDA Model : SPD-M40
Start Wavelength : 200 nm
End Wavelength : 700 nm
Cell Temp. : 40 C
Polarity : +

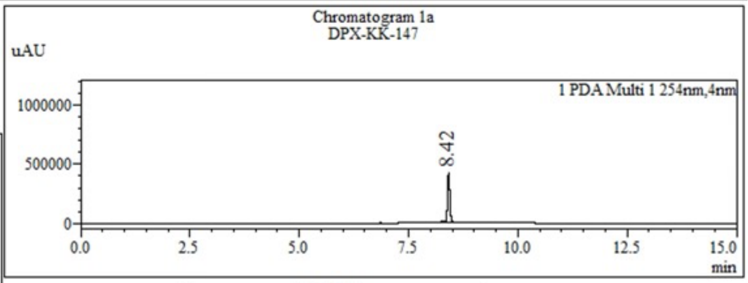

Peak Table

PDA Ch1 254nm

Peak#	Ret. Time	Area%
1	8.418	100.000
Total		100.000

Figure S22. HPLC chromatogram of **1a**

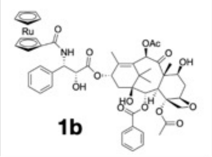

1b

Sample Information	
Sample Type	: Unknown
Sample Name	: DPX-KK-168
Sample ID	: DPX-KK-168
Vial#	: 2
Injection Volume	: 0.5

Method

<<LC Time Program>>

Time	Module	Command	Value
5.00	Pumps	B.Conc	90
6.00	Pumps	B.Conc	90
8.00	Pumps	B.Conc	30
15.00	Controller	Stop	

<<Column Infomation>>
Column Name : Kinetex XB-C18
Column Length : 50 mm
Inner Diameter : 2.1 mm
ParticleSize : 1.7 um

<<Mobile Phase Name>>
Mobile Phase A : water+0.01%HCOOH
Mobile Phase B : Methanol+0.01%HCOOH

<<Data Acquisition>>
LC Stop Time : 15.00 min

<<Pump>>
Total Flow : 0.3000 mL/min
B Conc. : 30.0 %

<<Autosampler>>

<<PDA>>
PDA Model : SPD-M40
Start Wavelength : 200 nm
End Wavelength : 700 nm
Cell Temp. : 40 C
Polarity : +

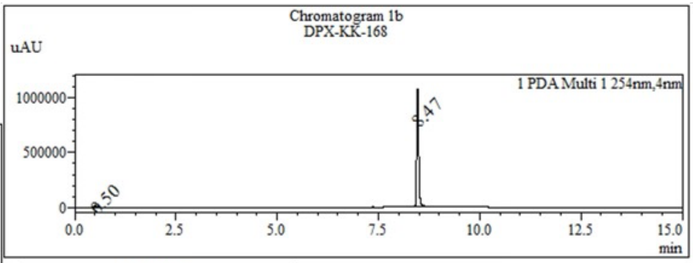

Peak Table

PDA Ch1 254nm

Peak#	Ret. Time	Area%
1	0.505	2.112
2	8.469	97.888
Total		100.000

Figure S23. HPLC chromatogram of **1b**

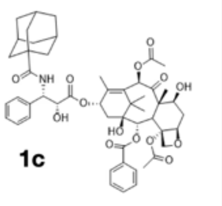

1c

Sample Information

Sample Type	: Unknown
Sample Name	: DPX-KK-187
Sample ID	: DPX-KK-187
Vial#	: 30
Injection Volume	: 0.5

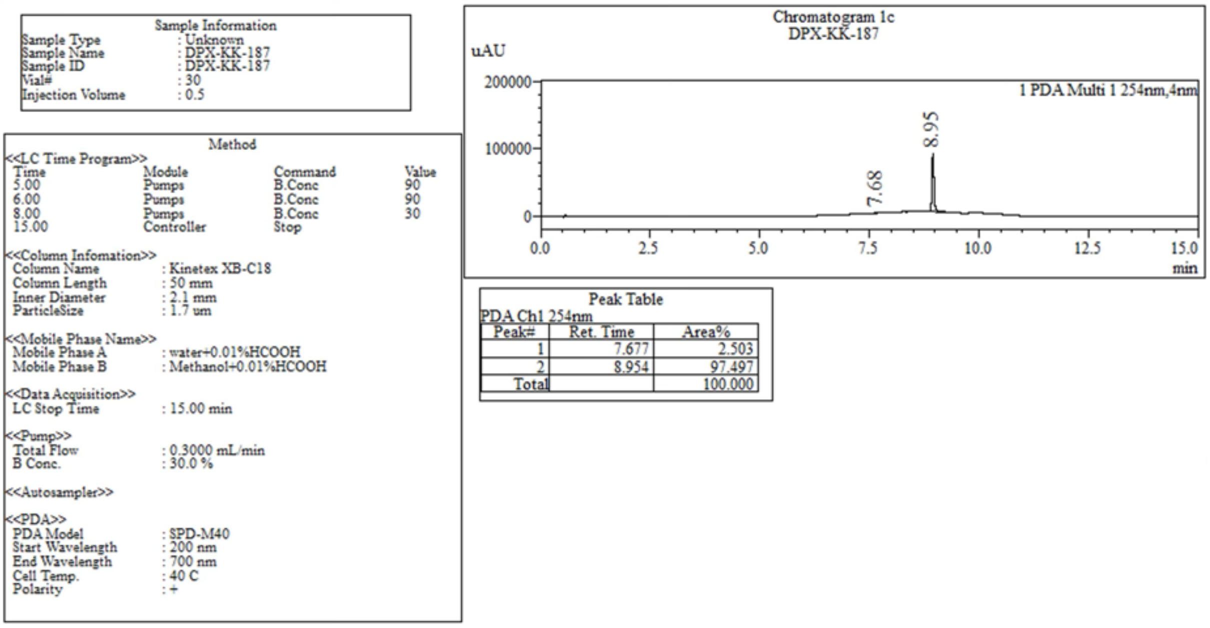

Peak Table

PDA Ch1 254nm

Peak#	Ret. Time	Area%
1	7.677	2.503
2	8.954	97.497
Total		100.000

Method

<<LC Time Program>>

Time	Module	Command	Value
5.00	Pumps	B.Conc	90
6.00	Pumps	B.Conc	90
8.00	Pumps	B.Conc	30
15.00	Controller	Stop	

<<Column Infomation>>
Column Name : Kinetex XB-C18
Column Length : 50 mm
Inner Diameter : 2.1 mm
ParticleSize : 1.7 um

<<Mobile Phase Name>>
Mobile Phase A : water+0.01%HCOOH
Mobile Phase B : Methanol+0.01%HCOOH

<<Data Acquisition>>
LC Stop Time : 15.00 min

<<Pump>>
Total Flow : 0.3000 mL/min
B Conc. : 30.0 %

<<Autosampler>>

<<PDA>>
PDA Model : SPD-M40
Start Wavelength : 200 nm
End Wavelength : 700 nm
Cell Temp. : 40 C
Polarity : +

Figure S24. HPLC chromatogram of **1c**
